# Small-angle X-ray scattering profile calculation for high-resolution models of biomacromolecules

**DOI:** 10.1107/S160057672500562X

**Published:** 2025-07-16

**Authors:** Kristian Lytje, Jan Skov Pedersen

**Affiliations:** a Department of Chemistry and Interdisciplinary Nanoscience Center (iNANO), Aarhus University, Denmark; DESY, Hamburg, Germany

**Keywords:** small-angle X-ray scattering, biomacromolecule solutions, excess scattering, hydration layers, excluded volume scattering

## Abstract

We introduce a new approach to calculating small-angle X-ray scattering (SAXS) profiles from high-resolution atomic structures, which is realized in the open-source software *AUSAXS*. We employ an efficient implementation of the Debye equation, incorporating both accurate excluded volume models and a novel hydration shell model based on explicit dummy atoms. Two new excluded volume models are presented: (i) a simple, heterogeneous equivalent atom model, and (ii) a grid-based model. The two approaches reduce the risk of overfitting by either eliminating fitting parameters or introducing a safer volume scaling method. These models are compared with the traditional Gaussian sphere method, which is widely used in existing software. The comparisons reveal significant shortcomings in previously accepted methods, suggesting they may be more prone to overfitting than previously thought. This underscores the importance of a well tested and openly accessible baseline implementation like *AUSAXS*. *AUSAXS* is freely available at https://github.com/AUSAXS/AUSAXS.

## Abstract 2

We present a new analysis approach for calculating the solution X-ray scattering of high-resolution models of biomacromolecules. We introduce novel grid-based methods for evaluating both the hydration shell and excluded volume scattering contributions. The methods are implemented in the new open-source software *AUSAXS*.

## 1. Introduction

Small-angle X-ray scattering (SAXS) has come a long way since its invention and theoretical formulation in the early 20th century. The advent of computers enhanced the technique, enabling geometric modeling and, more recently, the prediction and validation of experimental scattering profiles using high-resolution atomic models for a wide range of biomacromolecules in solution. Today, SAXS is routinely used to study molecules and their interactions under near-physiological conditions, thanks to its nondestructive nature and ease of use.

A common application of the technique is to evaluate the scattering profile of a molecular high-resolution structure in solution. The simplest approach is to implement the general scattering equations for an ensemble of randomly oriented particles in solution, as first described by Debye (1915 ▸). While this equation is trivial to implement by itself, there are three additional factors which must be considered, making any practical implementation significantly more involved: (i) the implementation must be efficient enough to support rigid-body refinement; (ii) a hydration layer at the surface of the molecule contributes significantly to the scattering and must therefore be included in the calculation; and (iii) the excluded volume of the molecule must be assessed, such that the excess scattering can be calculated. These three points are discussed further in the following, with reference to existing literature.

As mentioned above, the first challenge is efficiency: the Debye equation is an 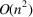 process in the number of atoms, making it an unattractive option for most implementations. To address this, several approximations have been developed: the spherical harmonics expansion proposed by Stuhrmann (1970 ▸), first used for high-resolution structures in *CRYSOL* (Svergun *et al.*, 1995 ▸) and later in other programs (Grudinin *et al.*, 2017 ▸; Liu *et al.*, 2012 ▸; Poitevin *et al.*, 2011 ▸), which in principle reduces the complexity to 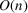; the hierarchical decomposition of the molecule, which transforms it to 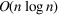 (Gumerov *et al.*, 2012 ▸); and distance binning in the Debye equation, which optimizes the serial evaluation of multiple *q* points, as used by Schneidman-Duhovny *et al.* (2013 ▸), Ravikumar *et al.* (2013 ▸) and Lytje & Pedersen (2024 ▸) and in the present work.

Secondly, as shown by Svergun *et al.* (1995 ▸), a structured layer of solvent molecules forms near the molecular surface. This layer has a higher scattering length density than bulk water and must therefore be accounted for when modeling the total scattering. However, owing to the complexity of this layer, determining its precise structure is analytically impossible, and it must thus be approximated.

To our knowledge, the best approximation of this hydration layer is obtained either through analytical integral theories such as RISM (Beglov & Roux, 1997 ▸) or using molecular dynamics simulations, which simulate the interactions between the molecule and solvent to generate a realistic hydration shell, as implemented in *WAXSiS* (Knight & Hub, 2015 ▸). Something similar but less accurate is used in *Fast-SAXS-pro*, where a pre-equilibrated water box is employed (Ravikumar *et al.*, 2013 ▸). An entirely different approach is to treat the solvent density as constant and implicitly include it by solving an extended Poisson–Boltzmann equation (Koehl & Delarue, 2010 ▸; Azuara *et al.*, 2008 ▸), as seen in *AquaSAXS* (Poitevin *et al.*, 2011 ▸). These three approaches are too slow for serial evaluations (Schneidman-Duhovny *et al.*, 2013 ▸), however, thus prompting the development of faster alternatives at the cost of precision.

*CRYSOL* and *SoftWAXS* model the solvent as a constant, fixed-width shell surrounding the molecule (Svergun *et al.*, 1995 ▸; Bardhan *et al.*, 2009 ▸). In contrast, *FoXS* implicitly accounts for the layer by attributing an additional contribution to the form factor of all atoms proportional to their solvent-accessible area (Schneidman-Duhovny *et al.*, 2013 ▸). *Pepsi-SAXS* uses an approach most similar to what we will present here, where the molecule is placed on a low-resolution grid (typically 3–4 Å) and dummy solvent molecules fill the unoccupied grid cells (Grudinin *et al.*, 2017 ▸; Steiner *et al.*, 2018 ▸; Baerentsen *et al.*, 2023 ▸). Most of these methods, except for *WAXSiS*, require an additional scaling factor to slightly correct the scattering contribution of the hydration layer (Svergun *et al.*, 1995 ▸; Grudinin *et al.*, 2017 ▸; Poitevin *et al.*, 2011 ▸; Schneidman-Duhovny *et al.*, 2013 ▸; Ravikumar *et al.*, 2013 ▸).

Thirdly, as previously mentioned, when molecules are in solution, only the excess scattering length density contributes to the measured signal. This excess can be evaluated by determining the excluded volume and subtracting its expected scattering from that of the protein. A brief review of previous approaches for modeling this volume is given below.

Most of the programs mentioned earlier follow the approach introduced by *CRYSOL* (Svergun *et al.*, 1995 ▸; Grudinin *et al.*, 2017 ▸; Poitevin *et al.*, 2011 ▸; Schneidman-Duhovny *et al.*, 2013 ▸; Ravikumar *et al.*, 2013 ▸), which is based on the method first described by Fraser *et al.* (1978 ▸). In this method, dummy atoms with Gaussian spheres are used to represent the excluded volume, with volumes dependent on the atomic type. Since these volumes vary with the chemical environment and molecular structure, standard values from the original measurement by Traube (1895 ▸) are commonly used, and a free fitting parameter is introduced to scale these volumes (Svergun *et al.*, 1995 ▸; Grudinin *et al.*, 2017 ▸; Schneidman-Duhovny *et al.*, 2013 ▸). *SoftWAXS* instead introduces a hierarchical cube approach somewhat similar to what we will present here, where the total excluded volume is determined by rolling a sphere along the van der Waals surface, thus ensuring non-overlapping atomic volumes.

In this work, we compare the methods which are still available with a baseline implementation in our new open-source program *AUSAXS* (*Aarhus University Small-Angle X-ray Scattering*). We introduce efficient methods for generating both the grid-based excluded volume and the hydration layer. We developed this program from scratch for two main reasons: first, to address our concerns about the fitting parameters used for rescaling the excluded volume scattering in existing software, which may lead to overfitting; and second, to have full control over the implemented methods to better optimize them for rigid-body optimizations, which will be presented in a future paper.

To compare these different methods, we use the concept of *partial* intensity profiles, which we define as the scattering contribution solely due to the molecular atoms, the solvent atoms or the excluded volume. Comparing these profiles, especially against those from molecular dynamics simulations, allows us to assess the accuracy of each method. As a final note, since these partial profiles are not always available by default, we strongly encourage future developers to make all these partial profiles, both the self- and cross-terms, accessible.

## 2. Theory

We will in this section develop the theory necessary for understanding our models.

### 2.1. Scattering equations

Using the first Born approximation, in the far field, the scattering amplitude is given as the Fourier transform of the electron density distribution ρ: 
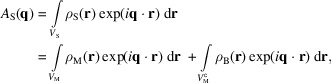
where the indices S and M refers to the entire sample volume and sum of molecular volumes, respectively, and B refers to the bulk density. The complementary volume 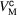 refers to everything not contained in *V*_M_. Continuing the derivation, by defining the bulk density as fluctuations about its average, 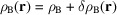 with 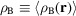, we can rewrite the second integral as
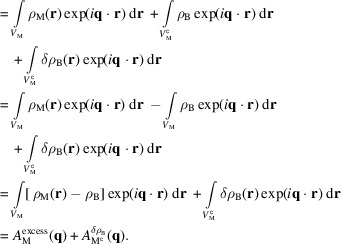
Here, we have used the fact that the integral of a constant density over the entire sample volume gives a delta function in *q*, which is experimentally unobservable. The first density 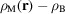, with 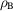 being the excluded volume contribution, is what is colloquially known as the excess density. Note that, when using this typical description of the total scattering, the excluded volume density is, by definition, completely homogeneous, as it represents the scattering from the average solvent density.

A scattering experiment does not measure the amplitude but rather the intensity, given as the square of this scattering amplitude: 
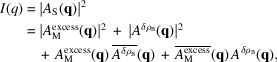
where the overline indicates complex conjugation. The two cross-terms cancel out, as the molecular and solvent fluctuation density distributions are uncorrelated. The second term is then typically removed through the background subtraction, where a separate measurement of just the bulk solvent is performed. This leaves only the first term, the excess scattering intensity, which is what we want to model here.

For *N* identical molecules uncorrelated in space and orientation, 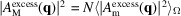, where 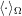 denotes the orientational average and 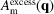 is the amplitude of a single structure representative of the ensemble. Though oligomers cannot be represented this way, they can still be modeled reasonably well by including a structure factor for the oligomerization (Baerentsen *et al.*, 2023 ▸; Larsen *et al.*, 2020 ▸). Performing the integral over orientations results in the Debye equation (Debye, 1915 ▸): 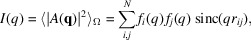
where 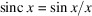 and the magnitude of 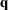 is defined as 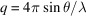, with 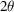 being the scattering angle and λ the X-ray wavelength. 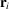 and 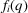 then denote the position and form factor of the *i*th scatterer, respectively. This equation is the foundation of our scattering models. To have more freedom in shaping the scattering power of individual atoms, we define 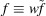, where 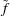 is the normalized form factor such that 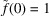 (henceforth used without the tilde). Since the weights *w* are then directly related to the number of electrons, this new form allows us to directly modify this number. With these, the Debye equation takes the form
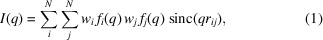
where, for X-ray scattering, the weight 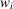 is generally given as the number of electrons of the scatterer, 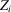.

#### 2.1.1. Hydration layer

The presence of the molecular surface will generally lead to higher solvent densities near the surface, which must be explicitly handled in any SAXS fitting routine. We have already given a brief description of how this step is handled by other programs and will thus focus on our own implementation here.

As our method is based on the Debye equation, we believe an explicit model of the hydration layer is the best approach since it is easily incorporated into the scattering equations. More specifically, our implementation will utilize dummy hydration atoms along with a free scaling parameter for their scattering power, 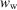. We can alter the original Debye equation to account for these by adding a sum over the dummy hydration atoms: 
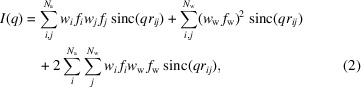
where we have suppressed the *q* dependence of the form factors 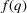 for simplicity. In this equation, 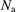 is the number of atoms in the protein and 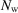 is the number of dummy solvent molecules. The subscript ‘w’ is short for ‘water’. We will later use the subscripts and superscripts ‘x’ and ‘a’ to denote the excluded volume dummy atoms and molecular atoms, respectively. To avoid confusing the entire biomolecule with the dummy water molecules, we will from here on refer to the entire biomolecule as a ‘protein’, although it may also be constituted of nucleic acids.

#### 2.1.2. Excluded volume

For the calculation of the excess scattering, the solvent volume displaced by the protein must be determined. Though there are a number of ways to deal with this, by far the most common approach is to use the formalism first proposed by Fraser *et al.* (1978 ▸), where dummy excluded volume atoms with Gaussian spherical form factors are superposed onto the atomic locations. Since the dummy atoms are centered on the real atoms, this amounts to adding a correctional term to the vacuum form factors: 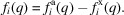
We define a Gaussian sphere as 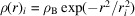, where 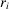 is the excluded volume radius of the atom, which will exclude a volume of 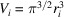 from the solvent. Performing the Fourier transform of this expression, we get the form factor of this excluded volume: 
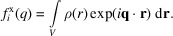
By performing this integral in polar coordinates, we can easily solve the two angular integrals, resulting in the following radial integral: 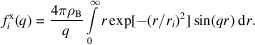
This is recognized as the Fourier sine transform of the function 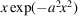, which has the standard solution
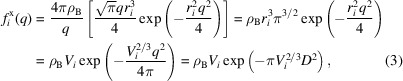
where we have used 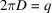 to obtain the final expression from the Fraser paper. Here, we also see that the volume of a Gaussian sphere 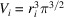 naturally appears in its form factor. We have explicitly derived the form factors using the more common 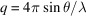 scattering vector magnitude, since there seems to be some confusion regarding its proper form in the literature. This is especially true since the derivation given in the original *CRYSOL* paper contains multiple significant errors or typos, as we will discuss later. Though we have verified that recent versions of *CRYSOL* are free of these errors, they continue to propagate through newer papers, indicating a need for this corrected derivation.

Since the excluded volume radii will in general depend on the chemical environment, *CRYSOL* (Svergun *et al.*, 1995 ▸) introduced the idea of adding a scaling parameter to all of the excluded volumes. Defining the scalable volume 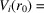

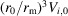
, we can factorize equation (3) as follows: 
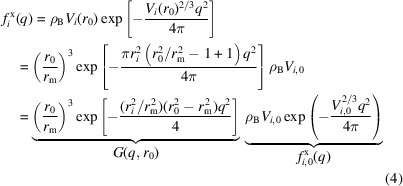
where 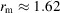
 Å is the average atomic radius and 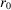 is the volume scaling factor, equal to 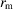 when there is no scaling. However, this form is inconvenient for actual calculations, since 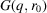 depends on the individual species through 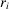. Because these excluded volume form factors are generally quite wide, we can remove this dependency by using the approximation 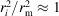. We thus end up with an overall adjustable Gaussian scale factor 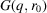 to adjust the excluded volume simultaneously for all atoms. Owing to its fitting power, this approach has been used in essentially all surviving programs since it was first adopted by *CRYSOL* (Svergun *et al.*, 1995 ▸; Grudinin *et al.*, 2017 ▸; Schneidman-Duhovny *et al.*, 2013 ▸).

#### 2.1.3. Optimizing for computation

We now want to optimize the Debye equation (1) for numerical evaluation. When accounting for both the explicit hydration layer and the excluded volume, it takes the final form
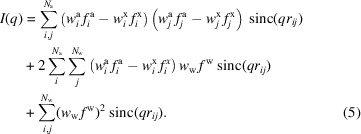
Note that, since we do not consider the hydration shell as a part of the excluded volume, 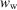 models its scattering relative to the bulk density. As previously mentioned, the primary idea of using Gaussian spheres to represent the excluded volume is that they are neatly incorporated into the scattering equations as a simple additional contribution to the atomic form factors. One could be tempted to do the same for the hydration shell – that is, define it in terms of the atomic positions. Indeed, this is exactly what is done in *FoXS* (Schneidman-Duhovny *et al.*, 2013 ▸) by defining 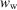 as a function of the solvent-accessible area. The surface atoms will then have a larger weight than those in the interior and are thus representative of the hydration shell. By further using an *equivalent atom* model, that is, defining all form factors to be equivalent such that 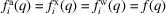, the Debye equation takes the especially simple form
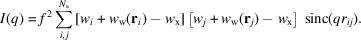
However, both of these approximations are likely too simple. In this model, the hydration shell overlaps exactly with the surface atoms, while in reality there is a small gap. This will in general lead to a shift in its scattering profile, as we will see later. The second approximation does not hold either, since the excluded volume form factors generally have a significantly stronger *q* dependence than the atomic form factors. We will thus primarily rely on equation (5) for our calculations.

Finally, since the intensity must typically be evaluated for hundreds of *q* points, the pure Debye equation is prohibitively slow because all pair distances and the 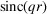 term must be recalculated for every such point. Thus, most implementations use a binning approximation, where all distance pairs are precalculated and binned into a distance histogram. Though determining the weights of all bins is still an 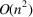 process, evaluating the intensity for a given *q* point is then linear in the number of bins. By also using predefined values of *q* (100 steps linearly distributed in the range [0.001, 1]) with cubic interpolation, the 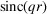 calculation can be converted to a simple table lookup.

## 3. Methods

In this section we will describe the major design decisions of our implementation and how it differs from existing methods.

### 3.1. Scattering of molecules in vacuum

The backbone of our implementation uses the binning approximation of the Debye equation (2). Normalized five-Gaussian atomic vacuum form factors from Waasmaier & Kirfel (1995 ▸) are used when explicit hydrogens are provided, though more typically the form factors of unified atomic groups from Grudinin *et al.* (2017 ▸) are used in combination with implicit hydrogens.

The default atomic electron count is assigned to each atom upon initialization and used as a weight for the Debye equation. When no explicit hydrogens are supplied, the number of covalently bound hydrogens is determined from the structure definition file, which is automatically downloaded from the RCSB Protein Data Bank (PDB; Berman *et al.*, 2000 ▸; https://www.rcsb.org/) using the residue name field of the PDB file. When such information is not available, either due to an incomplete PDB file or because of an unrecognized exotic residue name, the hydrogen-bond number is assumed to be zero and the atomic vacuum form factors are used instead. The N-terminus of each residue definition is specifically modified to have one less hydrogen bond since it will usually be part of a longer peptide chain. The hydrogen-bond number is then added to the scattering weight of each atom, which is modeled by the appropriate form factor accounting for the implicit hydrogens. When explicit hydrogens are provided, they are treated just like any other atomic species.

### 3.2. Hydration shell model

As previously mentioned, our hydration method is somewhat similar to that used by *Pepsi-SAXS*. By default, their method superposes a rough grid with cell sizes between 3 and 4 Å onto the protein, with a 12 Å padding in all directions. Each atom then occupies a spherical volume of radius 3 Å around it, thus excluding it from the hydration. All grid cells close to the surface and not already occupied by the molecular atoms are assumed to contain a solvent molecule, resulting in a highly ordered explicit hydration shell (Grudinin *et al.*, 2017 ▸).

Our method also overlays a grid on top of the protein, though we use a finer grid resolution of just 1 Å. The grid cells containing the molecular atoms are labeled as atomic centers and then expanded spherically using the van der Waals radius of the contained atom, marking all cells within range as occupied. Note how this expansion can be performed in linear time in the number of atoms, as any given real-space location can be mapped directly to its corresponding grid cell in constant time. Since the grid is so fine, this approach also gives a good approximation of the molecular surface, allowing us to use the grid to approximate the total molecular volume. After the grid cells containing the atomic centers have been expanded, the dummy solvent molecules are placed according to the following linear-time algorithm:

(1) Generate a number of radial lines from each atomic center. The default uses an angle of 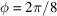 between each line, for a total of 26.

(2) Attempt to place a dummy solvent molecule at 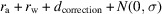 distance along each line. Here 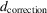 is a fixed corrective term, necessary since the hydration shell is typically closer to the molecule than the simple sum of the van der Waals radii of the atom *r*_a_ and the solvent molecule *r*_w_. 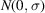 represents Gaussian noise, necessary to broaden the otherwise quite sharp solvent distribution.

(i) Generate another set of lines from the potential solvent location.

(ii) Mark each line at the following distances: 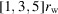.

(iii) Check each line mark for collisions with their corresponding grid cell. A weighted scoring heuristic is used to determine if the location is acceptable.

(iv) If the location is accepted, mark all grid cells within 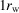 as occupied.

The idea of generating multiple radial lines from each atomic position is to ensure a more even solvent distribution across the molecular surface. This is ensured both by sampling many potential locations and through the simple scoring heuristic which determines if the environment is crowded; this is especially relevant inside cavities. The negative curvature of the surface means more locations are sampled within cavities than on the more positively curved outer surface, which will naturally lead to a higher density of solvent molecules if not corrected through *e.g.* our scoring heuristic. Note that the grid is used only for collision detection; the dummy solvent molecules can be placed at any continuous distance from the real coordinates of the atomic center.

**Figure 1:**
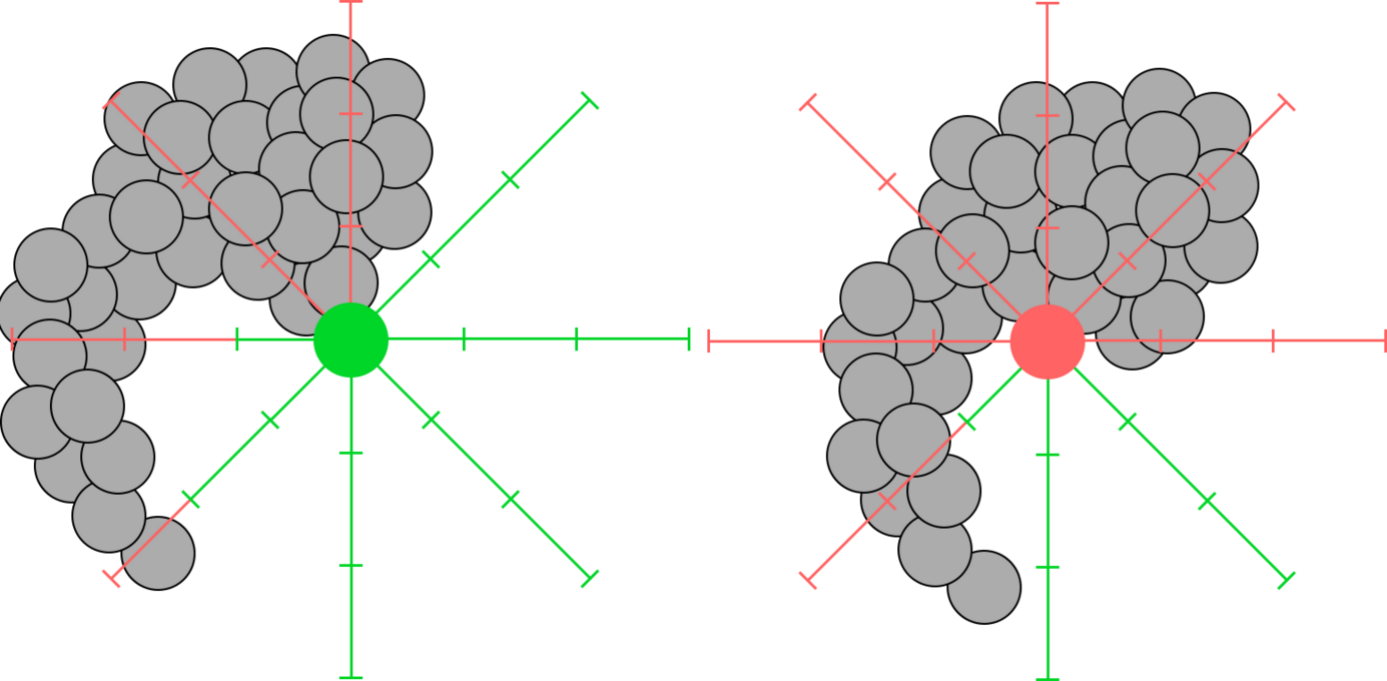
Two-dimensional illustration of the solvent placement algorithm. In the left panel, the proposed position is accepted, while on the right, it is rejected due to the crowded environment.

A 2D example of the algorithm is illustrated in Fig. 1 ▸, where the left solvent position is accepted and the right rejected. Since our simple algorithm does not account for the physics of the system, the solvent density is arbitrary and unrelated to the actual physics. To compensate for this, the hydration molecules are assigned a free parameter for scaling their scattering power to be optimized during the fitting. The main benefit of this approach is its efficiency: though checking each candidate solvent position requires some work, it is overall linear in the number of molecular atoms. An example of a full hydration shell generated by this method can be seen in the inset in the left panel of Fig. 3. Note that, since this is an empirical model, it is unsuitable for biomolecules which may have a varying solvent interaction strength across the surface, such as lipids.

### 3.3. Excluded volume model

The final task is to model the excluded volume, which turned out to be surprisingly complicated. For this reason, we have implemented not just one but three separate approaches for modeling it, to be able to compare them against each other. The first and simplest is a refinement of an older in-house method describing the excluded volume as identical for all atoms and located at the atomic positions (Baerentsen *et al.*, 2023 ▸). The second is equivalent to the Gaussian sphere model as championed by *CRYSOL* (Svergun *et al.*, 1995 ▸). The third and final model uses a novel grid-based homogeneous solvent similar to the one defined in and used by *SoftWAXS* (Bardhan *et al.*, 2009 ▸).

#### 3.3.1. Simple model

The first and default model supported by our program is also the most simple, hence its name. When the volume has been estimated by expanding the area around every atom in the grid cells, the total displaced number of electrons is calculated. These missing electrons are then equally distributed among all atoms by subtracting the average number of electrons per atom from their scattering weights; this is equivalent to placing the excluded volume on the atomic positions. This model is used in conjunction with the equivalent atom model also used by *FoXS*, such that 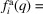

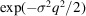
, with 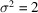
 Å, and 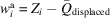, where 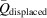 is the average number of electrons displaced per atom, in equation (2). Though this simple model lacks detailed structural information, it performs surprisingly well during testing, as we will see later.

#### 3.3.2. Fraser model

The second model is the Gaussian sphere model as defined by Fraser *et al.* (1978 ▸). We previously derived the corrected expression for the excluded volume form factors. This differs significantly from the literature, which is primarily based on the flawed derivation presented in the original *CRYSOL* article (Svergun *et al.*, 1995 ▸). There are three primary issues with their derivation:

(1) They use the hard-sphere volumes 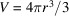 in place of the Gaussian volumes 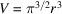. Note that this error will cancel as long as volume measurements are used as the basis for the radii, which is usually the case.

(2) The conversion from the crystallographic **D** scattering vector, as used by Fraser, to the vector **s** (= **q**) is not accounted for; see *e.g.* the last two expressions in equation (3). Though this error has propagated to multiple other articles, the authors usually correct it. We have explicitly verified that both *CRYSOL* and *Pepsi-SAXS* are unaffected by this, despite the error being present in both papers.

(3) There is a typo in the power of the volume term in their 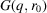 factor, where 3/2 has been used instead of 2/3. Though this is an obvious misprint, it has also propagated to newer articles (Schneidman-Duhovny *et al.*, 2013 ▸).

**Figure 2:**
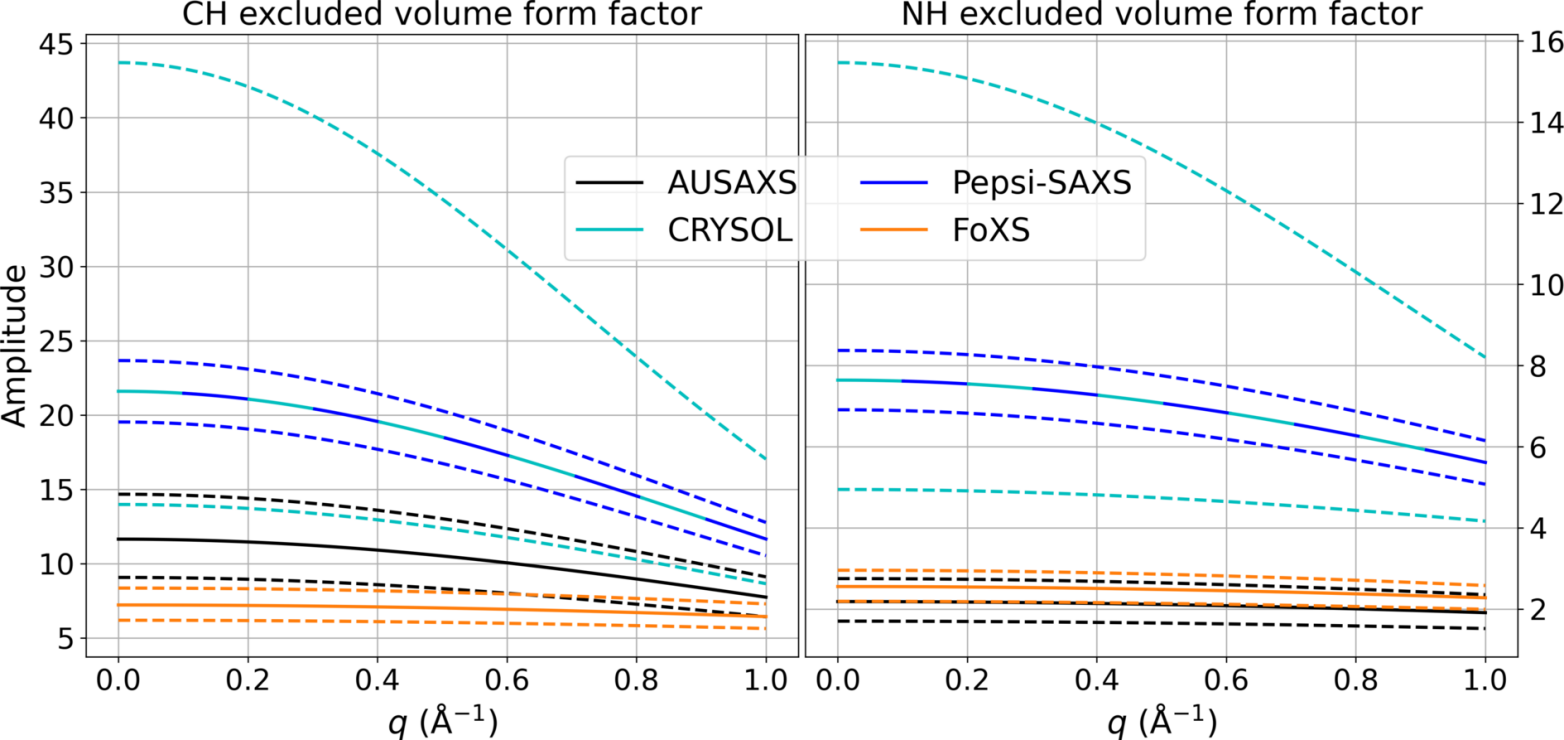
The excluded volume form factor of a CH and NH compound (solid lines), shown with the minimum and maximum possible variations on 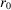 (dashed lines) as fitted by the different methods. *CRYSOL* scans the range 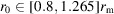, while the *AUSAXS* Fraser implementation uses 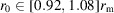. The other programs use the more limited range 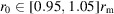.

As these three mistakes are continually propagating through the literature, we found it necessary to derive the correct expressions from scratch for posterity. To visually compare how each program models the form factors, Fig. 2 ▸ shows the Gaussian spherical excluded volume form factor of the CH and NH compounds, multiplied by the 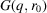 factors from each method for the minimum and maximum fitted 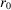 values. The figure also shows the form factor range from our Fraser implementation, which uses the minimum fluctuation volumes of Schaefer *et al.* (2001 ▸). This figure clearly shows how there are large differences between each method.

(1) *CRYSOL* fits a vast range of profiles, many of them obviously unphysical. The original article states that only the range 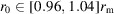 is used. Because of a change in philosophy, this range has been expanded considerably since then, as we have observed values in the range 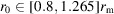. This corresponds to an assumed uncertainty of 26.5% in the radius. Such an uncertainty is obviously unreasonable, especially considering that it is not the radius which is the relevant parameter for the excluded volume form factor but rather the *volume*, which will then have a total uncertainty of 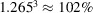. On the basis of informal talks with the current developer, we understand that this range was expanded to let the data guide the fit, with the intention that users would themselves reject unphysical values. Though the change was made with good intentions, without any emitted warnings when unphysical values are encountered, it is uncertain whether users react to this.

(2) It is clear that *Pepsi-SAXS* uses the same methodology as *CRYSOL*, though with a more reasonable 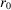 fitting range.

(3) The *FoXS* profiles cover a much smaller range, with essentially no dependency on the *q* axis. This clearly shows how *FoXS*’s equivalent atom approximation breaks down when used with the excluded volume form factors. More specifically, the assumed common exponential modulation 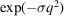 with 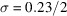
 Å^−1^ (see code excerpt S12 in the supporting information for this value) is too coarse an approximation of the excluded volume form factors. The problem is further exacerbated by some undocumented changes to the derived 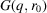 factor; see code excerpt S13 in the supporting information.

#### 3.3.3. Grid-based model

One major limitation of the Fraser model is that it introduces significant structure into the excluded volume when in reality it should be fully homogeneous, excepting internal pockets and cavities. To solve this, we have developed a new grid-based excluded volume method to model it homogeneously. The same 1 Å grid from the hydration model is used, such that finding the cell containing a given atom is a constant-time operation. The cells containing atomic centers are then spherically expanded again to a minimum radius of 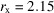
 Å to avoid small internal cavities, thus making the excluded volume approximately space filling. For clarity, note that each atom thus excludes solvent from a sphere of radius 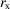 or 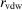, depending on which is larger. A dummy structure is then obtained by placing a dummy excluded volume atom in every marked cell, using a Gaussian sphere equivalent to the cell volume for the form factor. This is similar to what is proposed by and used in *SoftWAXS* (Bardhan *et al.*, 2009 ▸), where a hierarchical-cube approach is used to represent the excluded volume. The main difference is the Gaussian form factor and that they combine the individual excluded volume cells into larger cubes when possible to reduce the computational effort. Now, more than a decade later, improvements in modern consumer hardware have made such optimizations unnecessary.

Since the excluded volume approximation is space filling, there is no space between each dummy excluded volume atom and thus no possibility of scaling (fitting) the overall excluded volume of the protein as is done in the Fraser model. This is a major limitation as small corrections to these volumes are often required to obtain good fits. Since adding additional or removing any dummy atoms would necessitate an expensive recalculation of the entire distance histogram, we instead emulate the process by decomposing the scattering contribution of the total volume into an interior and exterior part. A free scaling parameter is then used to scale only the exterior contribution through the volume scaling method proposed by *CRYSOL* (Svergun *et al.*, 1995 ▸). The usual parameter bounds are relaxed for this model to effectively increase or decrease the surface layer width by 1 Å. A cross-section of the excluded volume generated in this manner can be found in the supporting information, Fig. S10.

An overview of our discussion so far can be seen in Table 1 ▸, where the methods used by each program are shown.

#### 3.3.4. Fitting

Table 2 ▸ summarizes the number of optimized parameters using each of the three excluded volume models. Though optimizing the excluded volume scaling is in principle not required for the Fraser method, practically it is, as we will see later. *a* and *b* denote the overall scale and background, respectively.

The model parameters, 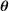, are estimated by minimizing the reduced 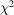 goodness-of-fit function 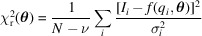
where ν is the number of degrees of freedom, that is, the number of free parameters in the model. Typically, a value 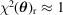 indicates a statistically good fit. Parameter optimization is carried out using the global_function_search algorithm from *Dlib* (King, 2009 ▸). Since the model intensity is computed for a fixed sequence of *q* values, a cubic interpolation algorithm is employed to obtain the intensity at the experimental *q* values.

## 4. Analysis

In this section we will take a closer look at all of our methods by comparing them with existing programs. To aid this analysis, we use the concept of *partial* intensity profiles. We define these as distinct parts of the total intensity curve, such that 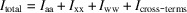. Here ‘aa’ is short for atomic–atomic, ‘xx’ (excluded volume)–(excluded volume) and ‘ww’ water–water. Comparing these intensity profiles from the different methods is not only an easy way to visualize the differences between them but also a powerful analysis tool. Since most of the programs mentioned in the *Introduction*[Sec sec1] are no longer available, with their home pages having been taken down years ago, we are here limited to comparing against *CRYSOL*, *Pepsi-SAXS*, *FoXS* and, to a more limited extent, *WAXSiS*.

### 4.1. Examining the hydration model

**Figure 3:**
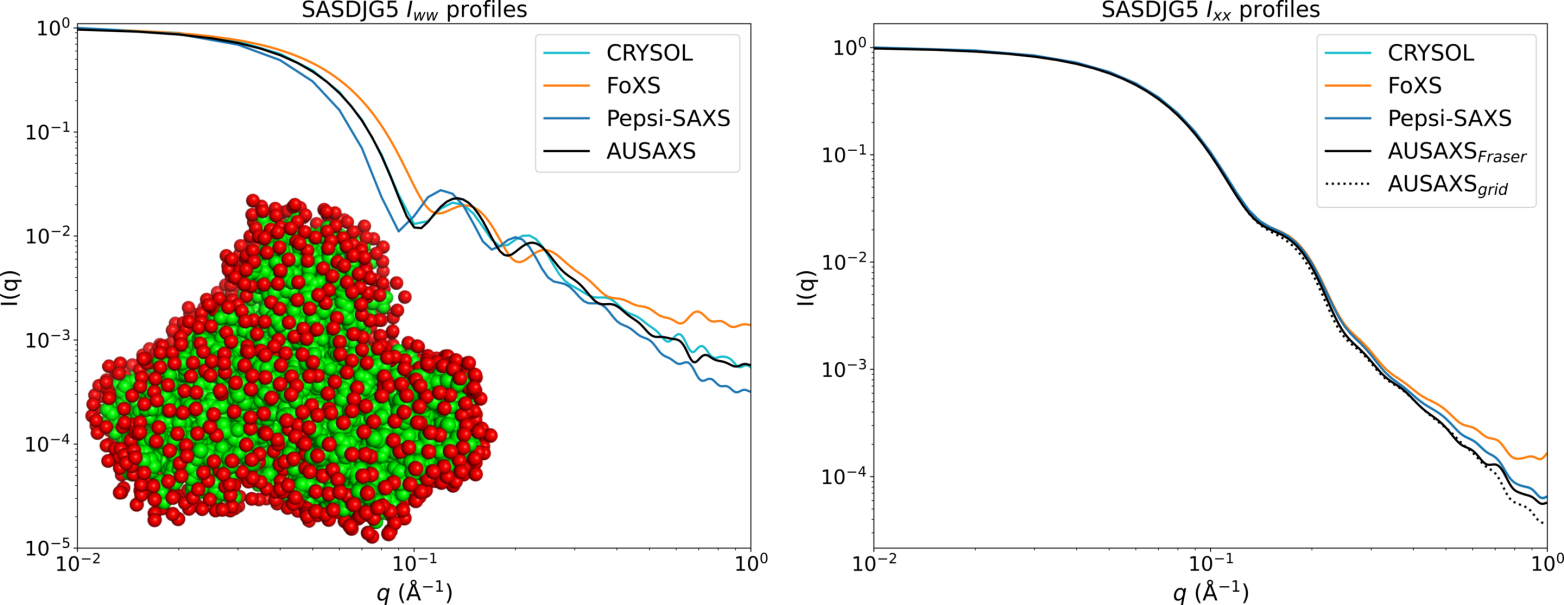
Partial profile comparisons for the hydration shell (left) and excluded volume (right). The inset in the left panel shows the hydrated structure for SASDJG5 obtained from our model. The calculated scattering profiles using *CRYSOL*, *Pepsi-SAXS* and *FoXS* are also shown.

An example of a hydrated structure is shown as an inset in the left panel of Fig. 3 ▸ for the SASDJG5 protein (Lee *et al.*, 2020 ▸). Such hydrated models are written to disk by default when using our program. The figure also shows both the corresponding partial hydration profile of this structure and its excluded volume scattering. These are compared with those of *CRYSOL* version 3.1.3, *Pepsi-SAXS* version 3.0 and *FoXS* version 2.21.0, calculated directly by their respective programs using the default settings. For *WAXSiS* it was not possible to obtain such profiles since it is based on a different formalism integrating molecular dynamics simulations.

The comparison in the left panel of the figure shows that our hydration model is quite similar to that from *CRYSOL*, though it is somewhat shifted with respect to the *Pepsi-SAXS* and *FoXS* profiles. This is probably due to the larger gap between the protein surface and hydration layer for the former, and the non-existent gap in the latter, where the scattering is approximated on the basis of the surface atoms. The right panel shows the excluded volume profiles, where *CRYSOL* and *Pepsi-SAXS* overlap exactly. Note that even minor differences in these profiles can significantly impact the quality of the fit.

**Figure 4:**
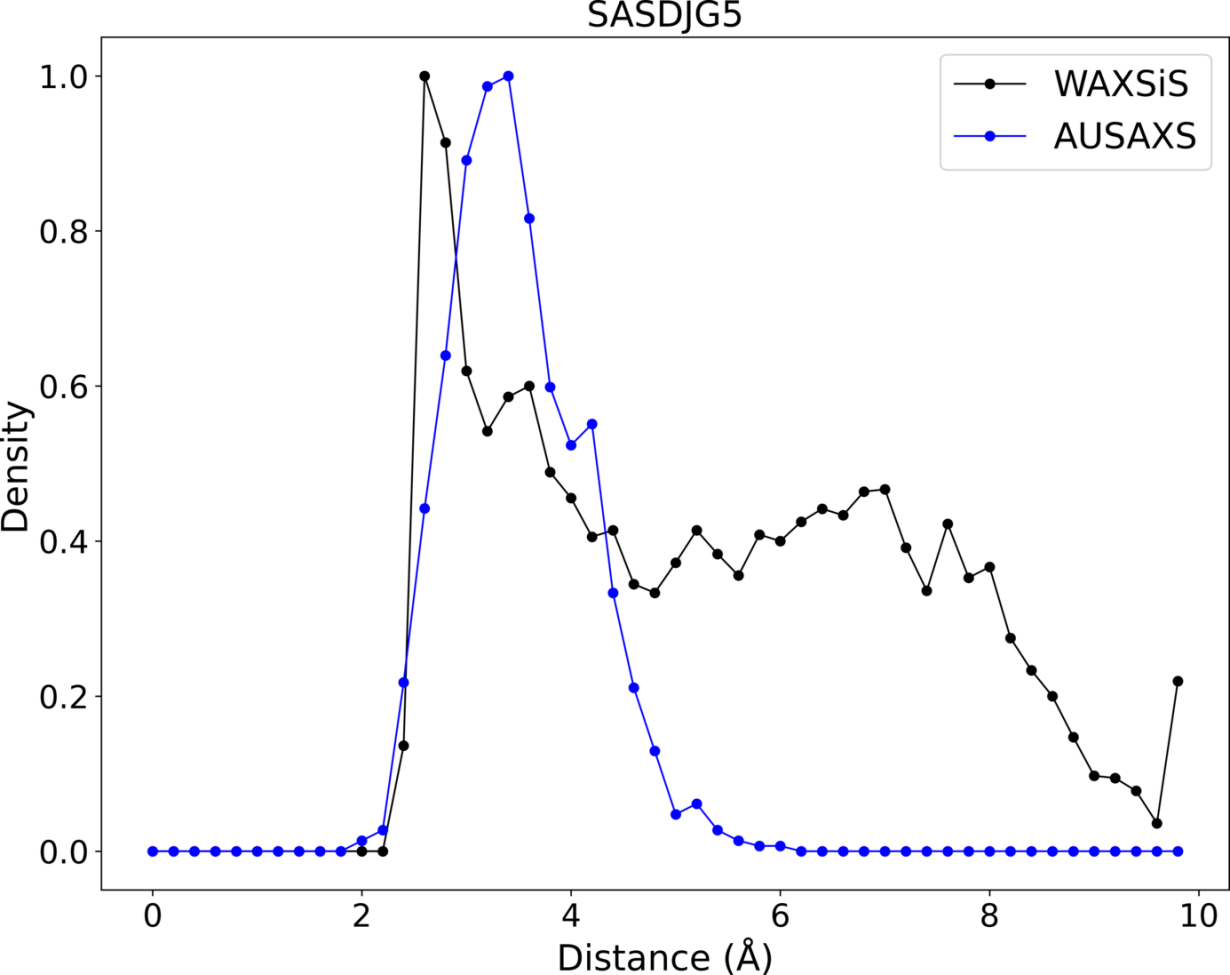
The solvent density distribution relative to the protein surface of *WAXSiS* and *AUSAXS*, where the latter has been calibrated to approximately reproduce the first peak.

Though it is theoretically possible to obtain the partial profiles from the *WAXSiS* molecular dynamics simulations, doing so is too involved and out of scope for this article. We instead compare against the distance distribution of the solvent relative to the protein surface. This distribution is trivial to calculate given the atomic positions from the final frame of the *WAXSiS* simulation, which is available by default from the *WAXSiS* webserver. Fig. 4 ▸ shows these distributions for both *WAXSiS* and our own hydration model, for which the values of both 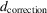 and the Gaussian sampling parameter 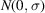 have been chosen to approximately reproduce the first *WAXSiS* peak. Note how the total *WAXSiS* distribution is significantly broader and has a visible second shell peaking at around 7 Å. We decided on a rather wide width because it is unclear exactly where the first hydration peak ends, and also because there is some variation in its shape; see the additional examples in Fig. S8 in the supporting information. Note also how our profile is in good agreement with the 3 Å width used in *CRYSOL*. The cutoff at high distances for the *WAXSiS* profile is due to the finite extent of the hydration envelope used in the program; see Knight & Hub (2015 ▸). Overall, this analysis indicates that our hydration method is in good agreement with established methods.

### 4.2. Examining the excluded volume models

We earlier presented the three excluded volume models which have been implemented in our software. To assess their performance against existing methods, we have made an extensive comparison using a semi-random selection of publicly available datasets from the Small Angle Scattering Biological Data Bank (SASBDB; Kikhney *et al.*, 2020 ▸). Most of the structures acquired in this manner turned out to be relatively globular and compact; see Table S2 in the supporting information. To ensure the comparisons are fair, some preprocessing of specifically the atomic structure files was needed. First, each file was simulated and fitted by the *WAXSiS* webserver, using the thorough-convergence option. The resulting structure file from this simulation was stripped of all explicit hydrogens, solvent atoms and ions and then used as input for the other programs. This step is necessary to avoid any potential bias towards the more commonly used programs and to avoid some of them making use of the additional information available in the simulated file.

The same versions of the three programs *CRYSOL*, *FoXS* and *Pepsi-SAXS* described earlier were used for this part. Beyond instructing each program to use the entire *q* range available in the dataset, no other special options were supplied. Typically this range is lower than 0.5 Å^−1^, though a few of the datasets go beyond this. Since *WAXSiS* correctly accounts for model uncertainties when averaging over multiple frames, it will naturally result in smaller 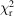 values than the other programs. Thus, to make the comparison more fair, we have recalculated their 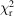 by linearly fitting their model profile to the data, using only the uncertainties in the data themselves. Note how this is equivalent to linearly fitting the data to the model as *WAXSiS* itself does (Knight & Hub, 2015 ▸). Also, most of the datasets are heavily oversampled in the high-*q* region, leading to artificially small 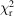 values in some cases.

**Figure 5:**
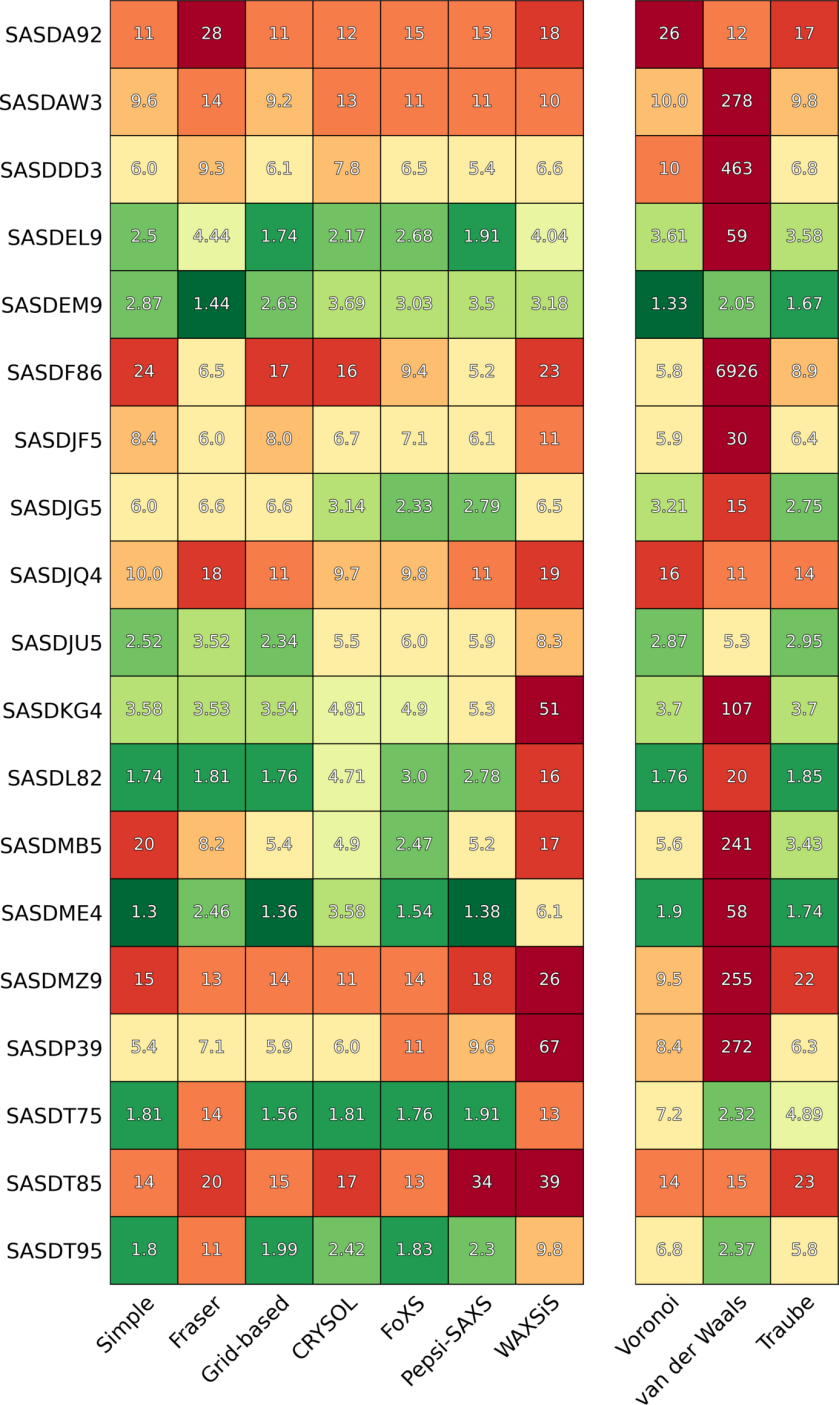
Comparison figure with 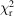 for a variety of structures. See the text for a detailed discussion. Another table containing examples with smaller variations between the methods is available in the supporting information, Fig. S1.

Fig. 5 ▸ shows the agreement determined for the various methods. The left table shows the goodness of fit 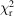 for all three of our methods, along with those obtained from *CRYSOL*, *Pepsi-SAXS*, *FoXS* and *WAXSiS* for a wide range of structures. Each square is colored according to the 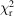 to visually aid the comparison. The right table shows three additional 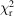 values obtained by using different excluded volume tables with our Fraser method. To reduce the size of the table, 21 additional examples were moved to Fig. S1 in the supporting information since the fit quality was very similar, with all 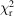 values being between 2 and 30, or with 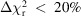 between all methods except *WAXSiS*.

These two tables contain a wealth of information. Starting with an internal comparison between our three methods, we see that the simple and grid-based methods generally give similar agreement with the experimental data. It is also clear that, excepting a few special cases, the Fraser method is the weakest of our models by a large margin.

Continuing to the external programs, even though it is based on molecular dynamics simulations, *WAXSiS* is the overall weakest method according to our recalculated 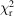 values. This is surprising as both its hydration shell and excluded volume model should be the most accurate. Regarding the different Fraser models, it is clear that ours is very different from the *CRYSOL*, *Pepsi-SAXS* and *FoXS* versions, which are all generally in agreement. To understand why, we have repeated the fits but using different excluded volume tables as the basis. Initially, we used the minimum fluctuation volumes of Schaefer *et al.* (2001 ▸), since these also assume Gaussian volumes for the atoms. However, in some cases, using the Voronoi tessellation volumes (Schaefer *et al.*, 2001 ▸; Pontius *et al.*, 1996 ▸), van der Waals volumes or Traube volume tables results in significantly improved fits, illustrating how the method has a significant dependency on the excluded volume table used as its basis.

We have categorized all structures in terms of their shape and mass in the supporting information, Fig. S2, to identify any possible correlations with the goodness of fit of the different methods and programs. No such correlations could be found, though this is possibly because we have an insufficient number of non-globular structures to compare against in our test data. We also tested the programs against complexes containing RNA in the supporting information, Fig. S9, though this comparison was also inconclusive.

#### 4.2.1. Examining the atomic volume dependency

To better understand this dependency on the underlying excluded volume table, we have performed Gaussian sampling within the listed uncertainties of the Schaefer volumes to generate a large series of random but consistent volume tables. This analysis is presented in the supporting information, Section S1.2, where we again find some models to be highly sensitive to the excluded volume table. The examples are mostly skewed Gaussian distributions of rather large widths, meaning even small variations in the excluded volume table can cause significant shifts in the 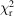 value. This is unfortunate as the atomic volumes are not known to a sufficiently high precision to avoid such fluctuations. This analysis is especially concerning in light of the other programs relying on volumes by Traube (1895 ▸), originally measured in 1895 with no listed uncertainties.

We have repeated this analysis using *CRYSOL* and found essentially the same result; see Section S1.2 in the supporting information.

#### 4.2.2. Examining the grid cell width

Note that it is not the method of using Gaussian spheres for the excluded volume form factors which is the issue in the Fraser method, but rather what excluded volume values to use for each atomic species and how they are distributed in space. This is an important distinction since we also use a Gaussian sphere for the dummy excluded volume form factor in our grid-based method. As the grid is space filling (and thus homogeneous), the excluded volume to insert in equation (4) is well defined, as it is simply the volume of a single grid cell. Since a Gaussian sphere is significantly larger than a solid sphere of equivalent volume, the form factors from neighboring cells will strongly overlap and together become a good approximation of the constant excluded volume density.

**Figure 6:**
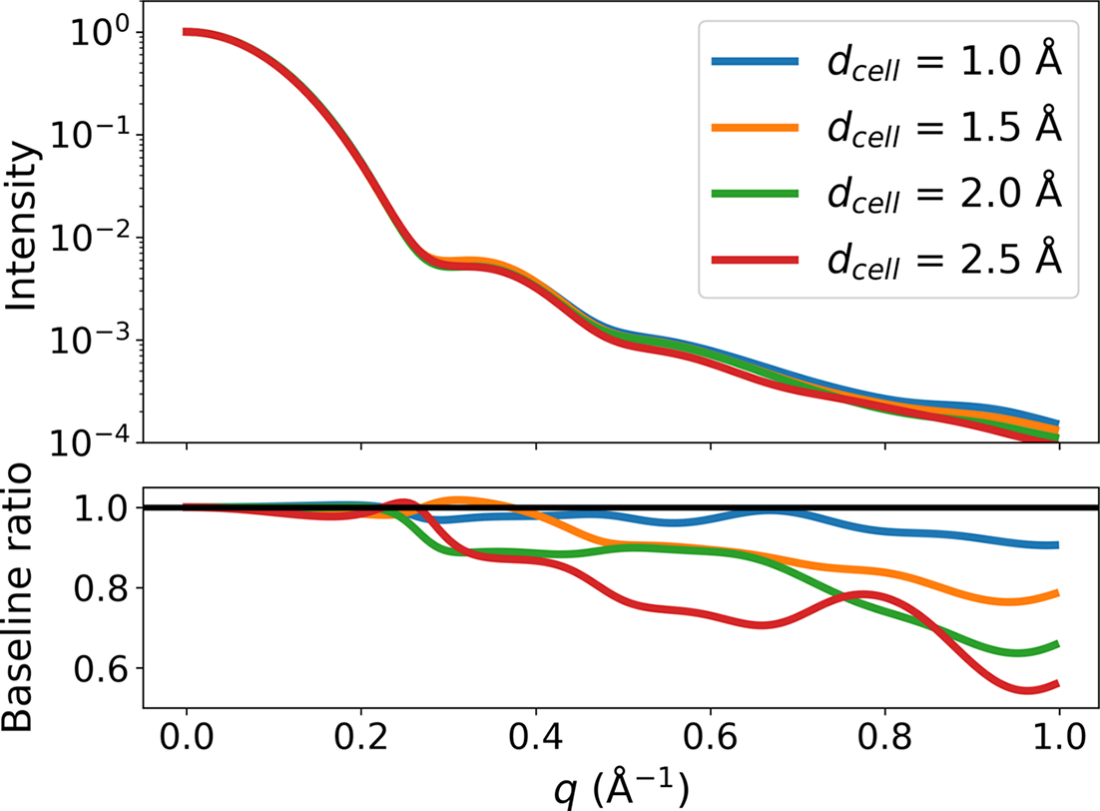
The excluded volume profiles for different grid resolutions 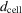. The top panel shows the normalized intensities, while the bottom shows the relative difference with respect to the model using 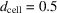
 Å. The deviations that occur when using larger radii can be somewhat mitigated by fitting the extension of the excluded volume.

The question is then what resolution is required for this grid to accurately represent the excluded volume. To determine this, we have calculated the excluded volume scattering for the same structure using different grid resolutions in Fig. 6 ▸. Here the ratios of the partial excluded volume profile obtained from using the indicated width and a reference width of 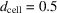
 Å are shown. On this basis, it is clear that even a conservative grid cell width of 1 Å is associated with substantial errors at high *q*. These errors are likely to be somewhat mitigated by fitting the size of the excluded volume in the actual program. As a result of computational limitations, 1 Å cell width is the smallest feasible option, which is thus the default in our program. However, this figure also shows that, for many systems where only data with *q* ≤ 0.5 Å^−1^ are measured, using 2 Å cells may be sufficient, and perhaps even desirable because of the factor 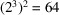 speedup in the Debye equation. Note that, since the grid is also used to estimate the molecular volume, adopting larger cell widths may cause both under- and overestimation of the volume due to the quantization. We therefore recommend using smaller cell widths for analyses where this volume is important. From the plot, it appears as though the relative intensity drops by about 10% for each increase in the cell width. It may be possible to introduce an empirical correction to the form factors to account for this drop. We plan to investigate this further in a future paper on rigid-body optimizations, where this potential performance benefit with respect to execution time will be more crucial.

### 4.3. Examining the impact of binning

**Figure 7:**
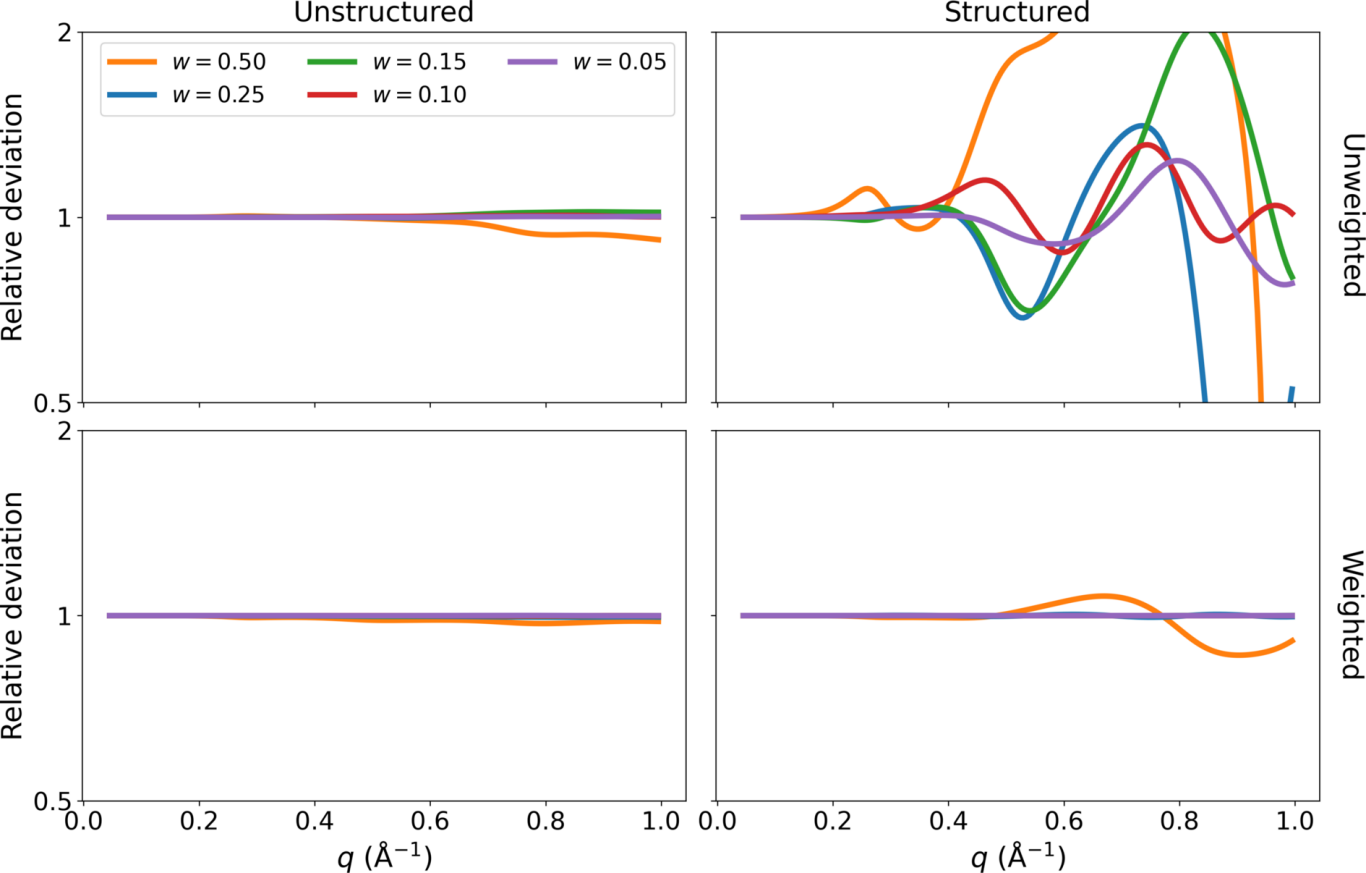
Comparison of weighted bins for the lysozyme structure PDB entry 2epe (M. D. Naresh, V. Subramanian, S. M. Jaimohan, A. Rajaram, V. Arumugam, R. Usha & A. B. Mandal, to be published). Profiles using regular (top) and weighted (bottom) bins in the Debye equation were calculated for two structures: a regular unordered protein (left), and a highly ordered grid approximation of the same protein (right). All profiles were divided by the true profile, calculated using the full Debye equation without binning.

To optimize the serial evaluation of multiple *q* values, we bin the calculated interatomic distances into a histogram, as already mentioned. Here, we examine the impact of such an approximation. There are two cases which must be examined: evaluating the scattering of a typical relatively disordered protein structure; and evaluating the scattering of a highly ordered crystal-like structure such as our grid-based excluded volume model. The latter requires significantly higher precision than the former. This leads us to the introduction of the *weighted binning* method, where the associated distance of each bin is shifted to the weighted mean of its contents. We compare both the two binning methods and the binning widths *w* in Fig 7 ▸. The large errors associated with using unweighted bins are surprising. Even for the disordered structure, an error of about 10% can be expected in the high-*q* region. This is probably due to the interatomic bond distances being overrepresented in the distance histogram, such that every single distance will propagate a small error to the overall scattering profile. Using weighted bin distances neatly solves this issue. On the basis of this analysis, we have chosen a value of 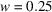
 Å as the width of our weighted bins.

### 4.4. Detailed examples

Having analyzed all major aspects of the implementations, it is now time to apply them to a few examples. We have already performed a major comparison in Fig. 5 ▸, so this section is dedicated to examining some of those examples in greater detail.

**Figure 8:**
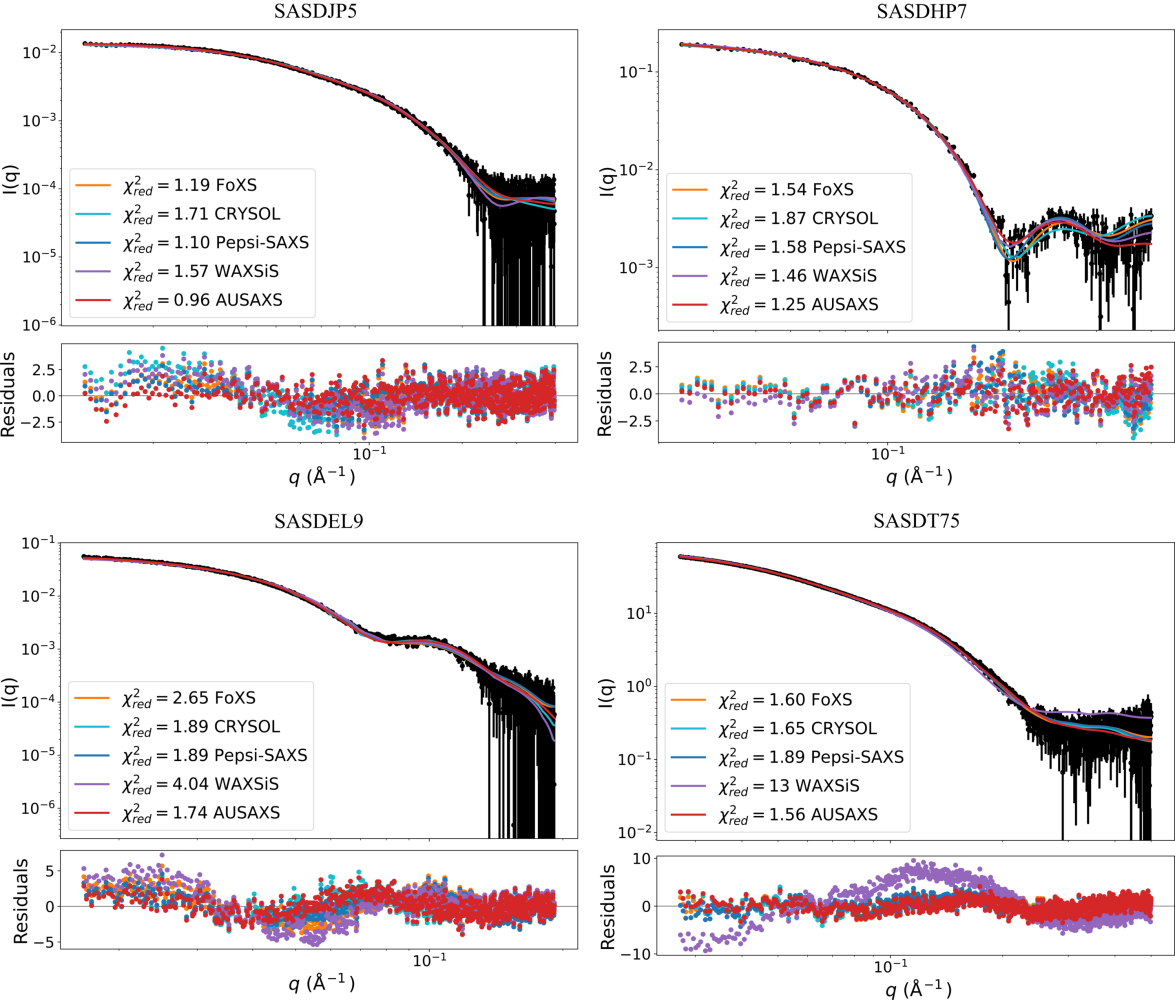
Randomly chosen fit examples from the comparison in Fig. 5 ▸. Both the calculated intensity profiles and their corresponding residuals are shown. SASDJP5 (Zhang *et al.*, 2021 ▸) is rod shaped, SASDHP7 (Markova *et al.*, 2020 ▸) globular, SASDEL9 (Sah-Teli et al., 2019 ▸) cage-like and SASDT75 (Sülzen *et al.*, 2024 ▸) rod shaped with loose loops.

Fig. 8 ▸ shows four randomly chosen examples from the large comparison table in Fig. 5 ▸, fitted using the grid-based method for *AUSAXS*. Note that the 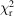 values here are somewhat different from those in the table, as they have been manually recalculated for all programs through a linear fit to the data. Though our grid-based method mostly comes out ahead, it is visually quite distinct from the other three methods; this is especially clear in the upper two figures. Generally, our method seems to be more accurate but with less variability in the high-*q* region, meaning it performs well on average but generally falls short when the data exhibit larger variation in this regime.

Our program is designed for analysis of typical SAXS data with a maximum *q* value of around 0.5 Å^−1^ (see *e.g.* Fig. 8 ▸). We have also analyzed the new consensus datasets (Trewhella *et al.*, 2024 ▸) with significantly larger *q* ranges, where a number of independent measurements have been averaged to obtain high-quality data with very low uncertainties. The analysis of these data again demonstrated that our grid-based method allows for less variability in the high-*q* region than the other programs. We believe that this is due to a too broad and slightly phase shifted excluded volume profile, leading to a damping of oscillations in this region. We have implemented several alternative excluded volume models in an attempt to solve this issue, such as fitting an overall expansion factor of the grid, thus effectively stretching it, and accounting for thermal vibrations by using an overall atomic displacement parameter (Moore, 2014 ▸) for both the atomic and dummy excluded volume atoms. Though the latter seems promising as an alternative to fitting the excluded volume, the fitted dis­place­ment parameters varied too much between different structures to be a meaningful parameter. An alternative answer could be to use normal mode vibrations (Panjkovich & Svergun, 2016 ▸), though we did not further investigate this possibility. We also implemented support for separately fitting the bulk solvent density, though we did not find the improvement in fit quality large enough to support introducing such a powerful additional fitting parameter. Both the two alternative excluded volume models and the bulk density fitting are available as non-default options in the program, though we do not recommend them for general use. See the online documentation for instructions on how to access them, as some of them may be hidden from the interface.

Another shortcoming of the grid-based method is the introduced orientational dependency of the calculated scattering. Since the grid axes are independent from those of the protein, when the protein is rotated, the grid will be slightly different, thus resulting in small variations in its scattering profile. This is further exacerbated by our hydration model also depending on the grid state, meaning it will also have an orientational dependency. Typically, this can be absorbed by the uncertainties in the measurement, but for the high-quality consensus dataset with its tiny errors, it cannot. When combined with the additional variation due to the Gaussian sampling in the placement of the generated dummy water molecules, this results in a total variation of about 30% in the 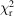 value for different orientations of the consensus data, while it was irrelevant for more typical datasets.

Finally, although the consensus SAXS data are of exceptional quality, there are no consensus *models*. Although a model is supplied with each dataset, there is no guarantee that the scattering from it should fit the data, as they were simply selected from the literature. Given all of these issues, we do not find it relevant to perform further analyses on the consensus datasets here.

### 4.5. Benchmarking the models

**Figure 9:**
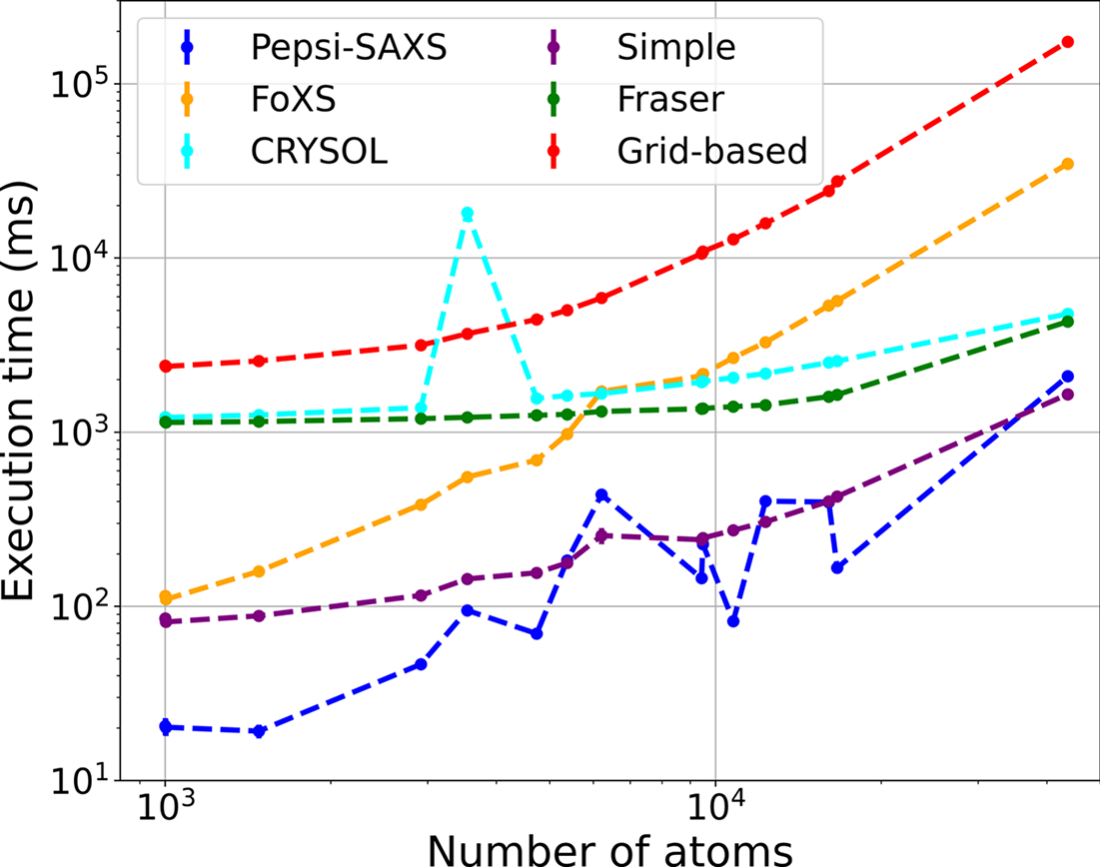
Benchmarking the different programs. The three lower entries shows the runtime of our three excluded volume models. This figure can also be found tabulated in the supporting information, Table S3.

As mentioned in the *Introduction*[Sec sec1], an important aspect of any application evaluating the solution scattering profile is its efficiency. Considering the large number of additional scatterers introduced by our grid-based excluded volume model, it is relevant to see how it compares against the typical programs. To this end, we have benchmarked all three of our models and compared them with the three other programs seen in Fig. 9 ▸. All programs were run in default mode, without any special arguments. *WAXSiS* is not a part of this comparison, since a typical thorough-convergence fit will take between 15 and 30 min on their webserver. The benchmark was carried out on a Linux laptop equipped with an Intel Core i7-1165G7 quad-core processor, thus supporting a total of eight concurrent threads with hyper-threading. From Fig. 9 ▸, it is clear that the underlying Debye calculations are very fast since the simple model is so competitive. This is a counterintuitive result considering the 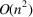 scaling of the Debye equation and contradicts the general consensus in the community. We believe that the explicit pair-distance calculations of the Debye equation make it more amenable to parallelization than the spherical expansion algorithm, which could explain the observed performance. See, for example, the article by Lytje & Pedersen (2024 ▸) for a benchmark of the single-threaded performance. Using the Fraser method roughly increases the runtime by a factor of five. Using the grid-based method further increases it by about one order of magnitude. Though this runtime is already manageable for most molecular systems, it can be dramatically reduced by using a lower-resolution grid, as already discussed.

## 5. Discussion and conclusions

We have already examined our novel hydration model in great detail and found that it is essentially equivalent to the implicit *CRYSOL* model. The primary benefit of using our explicit model, then, is that it can better model interior cavities, but at the cost of decoupling the solvent density from its physical value. While fitting the high-quality consensus data (Trewhella *et al.*, 2024 ▸), we noted that the fit quality is quite sensitive to the exact shape of the hydration shell, which is unfortunate considering the inherent variability due to the Gaussian sampling. This could potentially be solved by using more dummy solvent molecules in our model, or perhaps by also modeling the secondary density peak as seen in Fig. 4 ▸. The development of efficient but accurate hydration models could be a good avenue for future research, especially with the possibility of obtaining and fine-tuning the models against the highly accurate partial profiles from *WAXSiS*.

There is not much to say regarding the atomic profiles, as ours are practically identical to those obtained from most other implementations, as can be seen in Section S1.1 in the supporting information. The divergence at high *q* is curious, as our profile is systematically about 4% higher than the others. We have extensively checked and verified that this cannot be a result of numerical issues, meaning it is probably due to minor differences in the choice of atomic form factors, or perhaps issues with the convergence or orientational averaging of the spherical harmonics expansion.

While we have been able to reverse-engineer the base Gaussian spherical excluded volume form factors almost exactly, as can also be seen in Section S1.1 in the supporting information, we have been unable to replicate many of the fits obtained when using either of the other Fraser-based programs; see *e.g.* the ‘Traube’ column in Fig. 5 ▸. As the three other programs all have different hydration profiles as seen in Fig. 3 ▸ but still strongly correlate in their 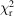 values, this is unexpected. However, as already mentioned, we have found for the high-quality consensus data that the interplay between not only the excluded volume and hydration profiles but also the atomic profile can be important for the fit quality. Thus, this can perhaps be explained as arising from the minor differences in each of the three profiles in our methods. Another possibility is that *CRYSOL* may be using a slightly different expression from equation (4) as found by Ginsburg *et al.* (2019 ▸), though we found this altered expression to negatively affect the comparisons. Without access to the *CRYSOL* and *Pepsi-SAXS* codebases, this issue is impossible to investigate further.

Regarding our own excluded volume models, it is clear that the grid-based method is superior. Not only does it give better results on average, it also requires less, if any, fitting to do so. Though we have only shown the 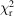 grid values including surface scaling in Fig. 5 ▸, when this scaling is disabled, it gives essentially the same results as the ‘Simple’ column, as can be seen in Fig. S2 in the supporting information. On the other hand, the Fraser method essentially always requires some amount of volume fitting to give reasonable values.

Beyond this, we have also shown in Section S1.2 in the supporting information that the Fraser model itself is prone to both over- and underfitting due to its strong dependence on poorly defined excluded volume measurements. Even the minor variations consistent with the listed uncertainties of the Schaefer *et al.* (2001 ▸) tables result in 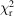 distributions of significant widths, indicating that a given structure can potentially be both accepted and rejected solely on the basis of the choice of excluded volume table. Considering that the uncertainties in the Schaefer volumes are generally around 10% and that the typically allowed uncertainty is 16% (5% in the radius), this indicates that the Fraser method may not be as robust as previously thought.

As an aside to this discussion, it is worth mentioning that Schaefer *et al.* (2001 ▸) provide two versions of the minimum fluctuation and Voronoi excluded volume tables: one explicitly including hydrogens, and another where they are assumed to be independent atoms. Though the two tables are similar, the volumes of all other compounds are generally smaller in the former to make room for the hydrogens. As we have already discussed, even small variations in these tables can dramatically affect the fit quality. For this reason we have implemented both versions of the tables, and automatically switch between them depending on if implicit or explicit hydrogens are used. Another question with these volumes is then which table to use: the minimum fluctuation or the Voronoi volumes. Schaefer shows that the minimum fluctuation volumes are generally more context dependent than the Voronoi volumes, meaning they depend more strongly on the size, solvent accessibility and number of contacts. However, since the minimum fluctuation volumes are also defined using Gaussian spheres for the atoms, it is unclear which set is the best default. We eventually decided on the minimum fluctuation volumes, though the Voronoi tables are also available through a simple switch. Yet another related discussion is how much the fitting parameter 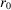 should be allowed to vary. Schaefer found the calculated Voronoi volumes to vary by about 20% between native and unfolded structures. Since we use the less stable minimum fluctuation volumes by default, we settled on using the maximum Voronoi volume variation of ∼25%, leading to 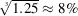 uncertainty in the radius.

A small caveat to this discussion is that we also found the choice of excluded volume model to only be relevant for a small subset of structures. In most cases, the results obtained from using the different models are almost equivalent (both in Fig. 5 ▸ and in Fig. S1 in the supporting information). The main benefits of the grid-based model, then, are that it gives more physically meaningful results even without the excluded volume scaling factor and that it has no dependence on poorly defined excluded volume measurements.

In an attempt to avoid these issues while keeping the performance benefits of the Fraser method, we introduced a new simple model describing the excluded volume as being evenly distributed on all atoms. As seen in the large comparison in Fig. 5 ▸, this method will typically give similar results to the far slower grid-based model, and with less possibility of overfitting since it does not perform any sort of excluded volume fitting. Note specifically how it is usually consistent with the grid-based 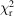 values without surpassing them. Though this may initially seem surprising, it is really just confirming that the excluded volume model is rarely important for the fit quality of typical SAXS data. Because of these strengths, we have chosen this as the default model for our software. When it does not prove sufficient, the user is free to switch to a more detailed excluded volume description instead.

We have here introduced our new software package *AUSAXS* for small-angle X-ray scattering analyses, its novel hydration shell model and the three supported excluded volume models. Our analysis indicates that, of these three models, the grid-based one is preferred to avoid overfitting. The program and its methods are implemented in modern C++20 and are both open source and freely available on our public GitHub repository https://github.com/AUSAXS/AUSAXS. The codebase was designed with efficiency and expandability in mind, with the hope that it can serve as a foundation for future community extensions and developments. Precompiled binaries for all major platforms are also available both as a command-line program and as a simple graphical interface, with accompanying online user guides. Discussions and contributions are more than welcome there.

## Supplementary Material

Supplementary information. DOI: 10.1107/S160057672500562X/yr5152sup1.pdf

## Figures and Tables

**Table 1:** Overview of the methods implemented in various programs

Program	Vacuum scattering	Hydration layer	Excluded volume
*AUSAXS*	Debye	Grid-based explicit atoms	Simple/Fraser/grid-based Gaussian spheres
*CRYSOL* (Svergun *et al.*, 1995 ▸)	Spherical harmonic expansion	Constant, fixed-width shell/explicit atoms	Fraser
*FoXS* (Schneidman-Duhovny *et al.*, 2013 ▸)	Debye	Solvent-accesible term in form factor	Fraser
*Pepsi-SAXS* (Grudinin *et al.*, 2017 ▸)	Spherical harmonic expansion	Grid-based fixed-width explicit shell	Fraser
*WAXSiS* (Knight & Hub, 2015 ▸)	Time-averaged amplitude	Simulated	Simulated
*D+* (Ginsburg *et al.*, 2019 ▸)	Reciprocal-space grid	Grid-based shell	Fraser/grid-based
*AquaSAXS* (Poitevin *et al.*, 2011 ▸)	Cubature	Poisson–Boltzmann equations	Fraser
*Fast-SAXS-pro* (Ravikumar *et al.*, 2013 ▸)	Pseudo-particle Debye	Pre-simulated water box	Fraser
*SASTBX* (Liu *et al.*, 2012 ▸)	Zernike polynomials	Constant, fixed-width shell	van der Waals volumes
*SoftWAXS* (Bardhan *et al.*, 2009 ▸)	Numerical quadrature	Constant, fixed-width shell	Grid-based hierarchical cubes

**Table 2:** Overview of the free parameters used in our methods Parameters marked with a ‘×’ must always be optimized. Parameters marked with a ‘/’ are optional.

Description	Parameter	Simple	Fraser	Grid
Linear fit	*a*, *b*	×	×	×
Hydration shell scattering scale	*c*	×	×	×
Excluded volume scaling	*d*		×	/
