# Supplementary material for: Small-angle X-ray scattering profile calculation for high-resolution models of biomacromolecules
Source: J Appl Crystallogr. 2025 Jul 16;58(Pt 4):1332–46. doi: 10.1107/S160057672500562X (PMC12321023; doi:10.1107/S160057672500562X)
Supplement: Supplementary file 1 [file j-58-01332-sup1.pdf]

JOURNAL OF APPLIED CRYSTALLOGRAPHY

Volume 58 (2025)

Supporting information for article:

Small-angle X-ray scattering profile calculation for high-resolution models of biomacromolecules

Kristian Lytje and Jan Skov Pedersen

Journal of Applied Crystallography

ISSN 1600-5767

Supplementary information: AUSAXS: an open-source small-angle X-ray scattering profile calculator for high-resolution models of bio-macromolecules

Kristian Lytje[1] and Jan Skov Pedersen [1]*

[1]Department of Chemistry and Interdisciplinary Nanoscience Center (iNANO), Aarhus University. Correspondence e-mail: jsp@chem.au.dk

This is the supplementary information providing additional details and resources to support the main article. Included are additional fit examples, χ_r^2 distributions when varying the excluded volume tables, additional solvent distance distributions, a program flowchart, and code excerpts from the `FoXS` source code.

1. Supplementary information

1.1. Implementing the external excluded volume models

Since the expressions for the excluded volume form factors of `CRYSOL`, `Pepsi-SAXS`, and `FoXS` are all somewhat different from our own, we have attempted to reverse-engineer their implementations in our own software to compare and understand the differences. Though these programs have made all of the partial profiles available, only the unscaled excluded volume profile can be obtained, that is, without the $G(q, r_0)$ factor, which we have thus been unable to verify.

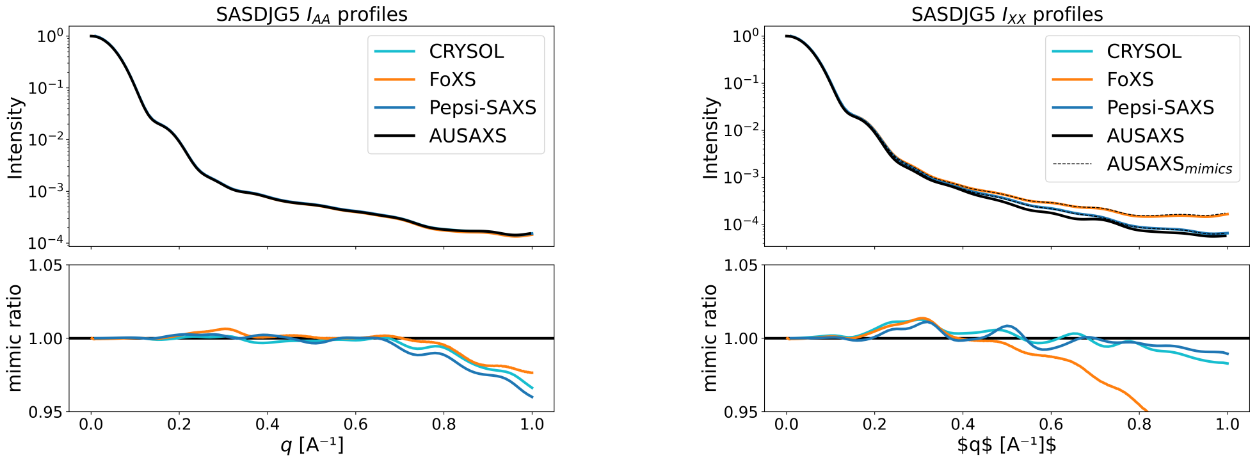

Figure 1
Profile comparisons with other programs. The left panels shows a comparison of the partial atomic-atomic profiles, which should always be identical. The top right panel shows the partial (excluded volume)-(excluded volume) profiles which are unique for each program. The bottom right panel shows the 'mimic ratio', that is, the ratio of the curve from the program itself and our implementation of their method. Such a comparison is necessary to validate the methodology, as inaccuracies are present in all three articles.

First the atomic partial profiles are evaluated and compared for the sample *SASDJG5* structure. Since they depend only on the atomic positions and the vacuum form factors, only minor deviations are to be expected. These profiles are shown in the left panel of figure 1 where indeed only a minor difference in the high-q region can be seen, which is likely due to slight differences in the vacuum form factors. Next we compare the excluded volume profiles, which can be seen on the right panel of the same figure. Again, the ratios are nearly constant, indicating that *our* implementation of *their* methods must be correct. Note that we are only comparing the unscaled excluded volume profiles since the $G(q, r_0)$-scaled curves are not available from any of the other programs. Based on this, it is apparent that both `CRYSOL` and `Pepsi-SAXS` correctly accounts for the $q = 2\pi D$ conversion of the scattering vector even though it is not present in their articles. This is important as this factor dramatically alters the excluded volume form factor profiles. Though `FoXS` explicitly included the conversion factor in their derivation, a closer examination of their open-source codebase reveals that they somehow still failed to account for it; see the code excerpt S13.

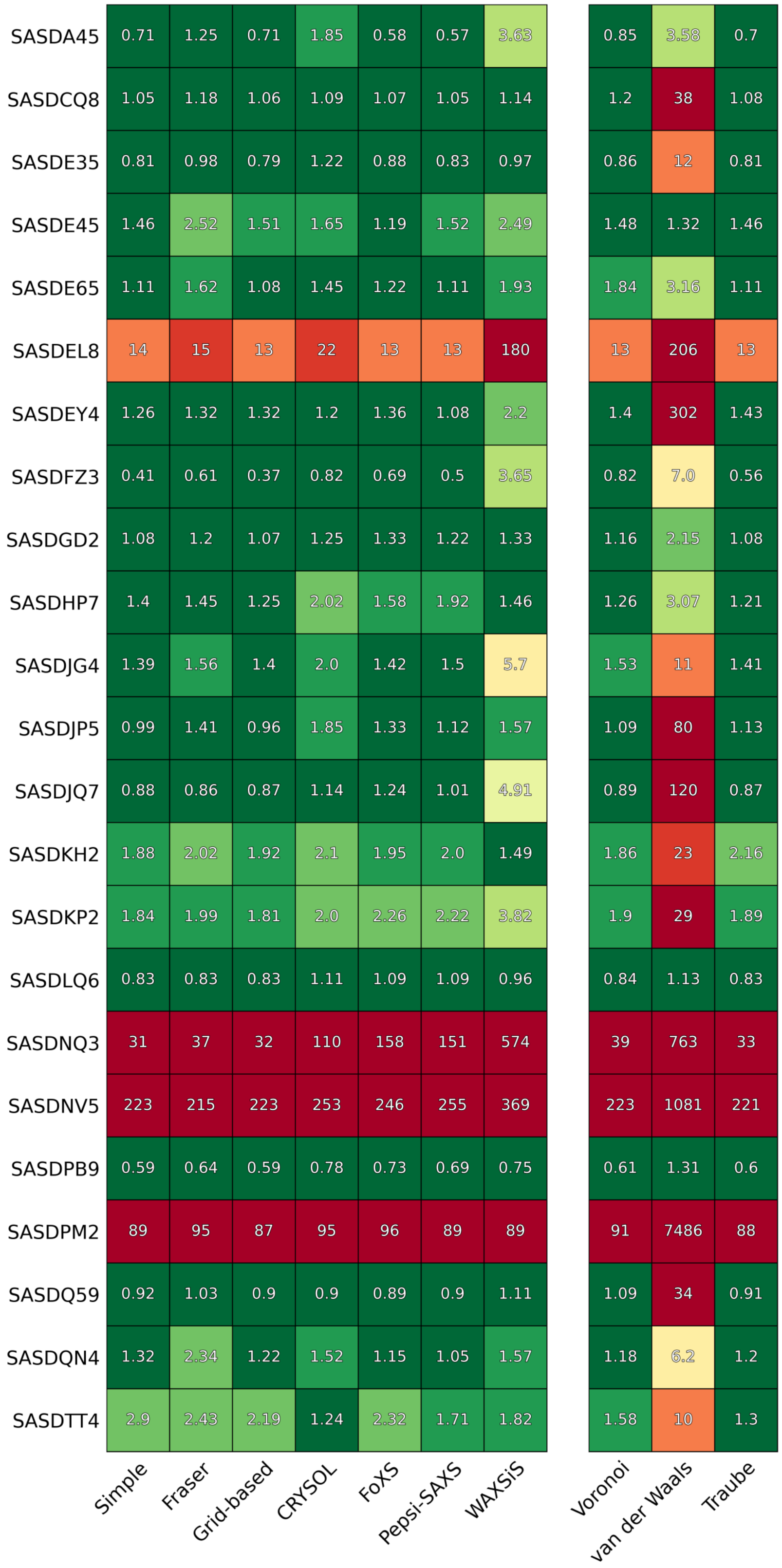

Figure S1
Comparison figure. This contains all the examples which were skipped in the main text for being too similar (both good and poor) in fit quality.

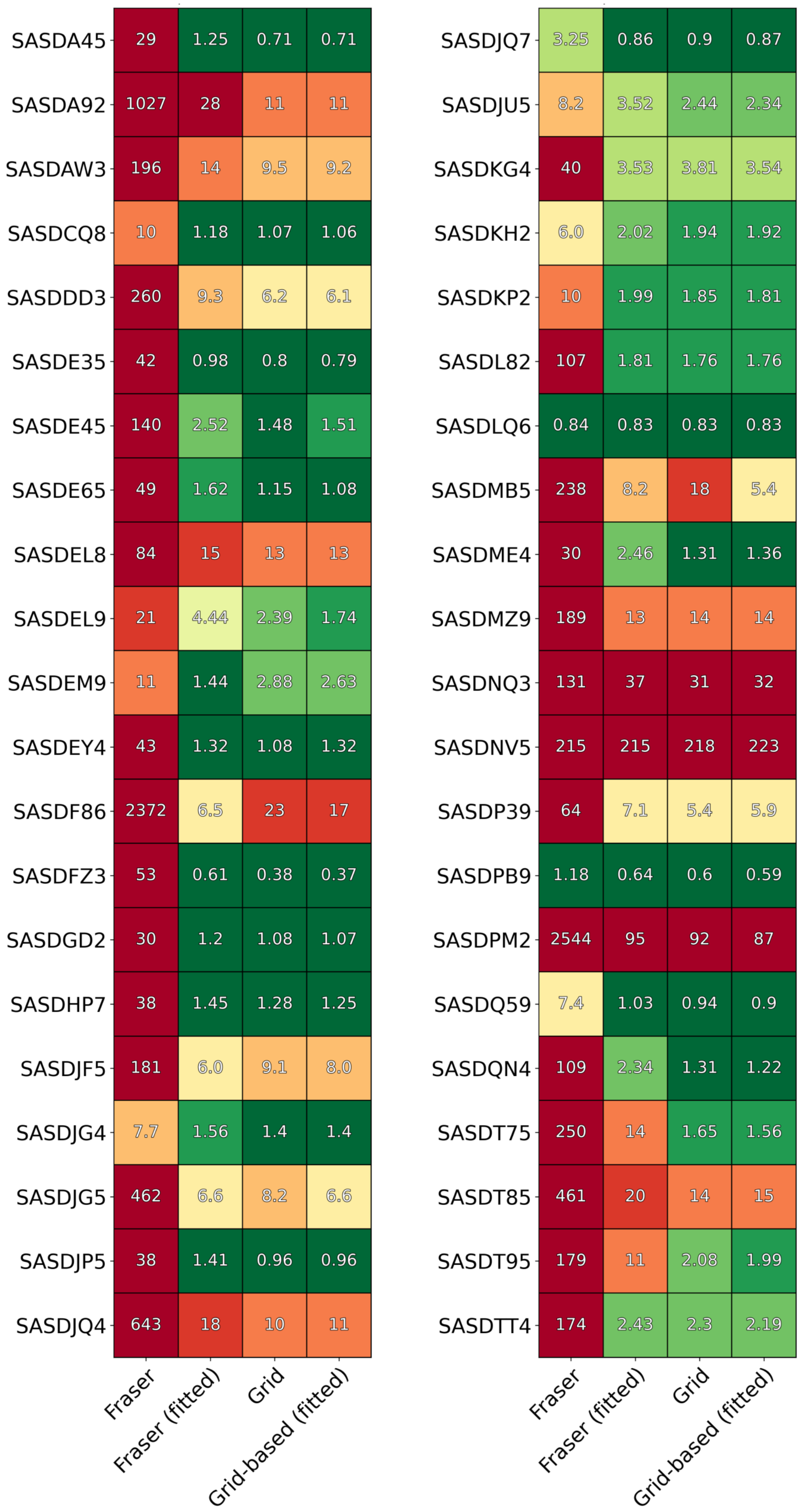

Figure S2

Comparison figure. This shows the difference in fit quality when enabling the excluded volume fitting. Note how the grid-based method produces far better results even without fitting.

1.2. Excluded volume table distributions

To better understand the strong dependency on the excluded volume tables discussed in the main text, we have performed Gaussian sampling within the listed uncertainties of the Schaefer volumes (Schaefer *et al.*, 2001) to generate a large series of random but consistent volume tables. Using these with our implementation of the Fraser model, including the excluded volume fitting, results in the χ_r^2 distribution seen in figure S3. Note the large widths of both distributions, illustrating how even minor changes to the table can have drastic consequences for the goodness-of-fit χ_r^2 value.

The bottom panel of these figures also shows how the χ_r^2 value correlates with the volumes of each atomic species. In all cases, the carbon volumes have a strong influence on the χ_r^2, which is unsurprising considering how many carbons are present in the backbone.

In figure S7 the analysis have been repeated but using `CRYSOL` instead of `AUSAXS`. Their *SASDJG5* distributions are significantly smaller than ours, and have better average goodness-of-fit values. This could potentially be due to also fitting the solvent density, as we have observed this can dramatically improve the fit quality in our own program. The second example for the *SASDJY3* structure agrees with our findings, as it also shows a wide Gaussian distribution.

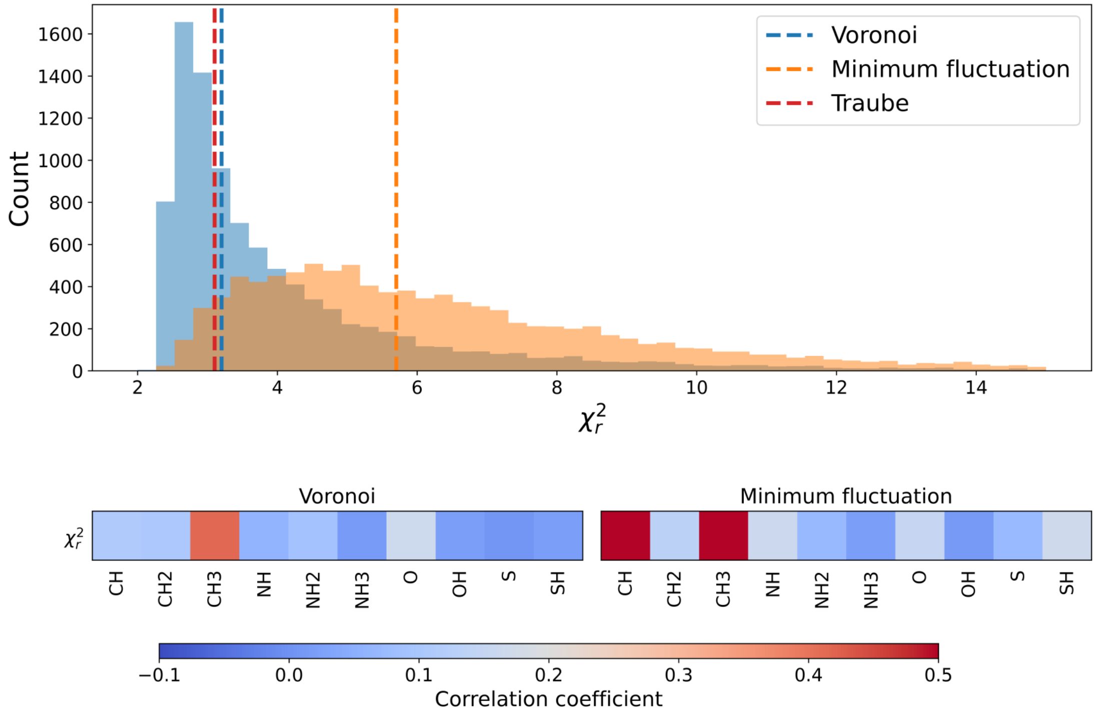

Figure S3
SASDJG5: The top panel shows the χ_r^2 distribution when randomly sampling excluded volume tables within the uncertainties reported by Schaefer *et al.* (Schaefer *et al.*, 2001). The dashed line corresponds to the mean values. The bottom panels show how each volume entry correlates with the χ_r^2 value.

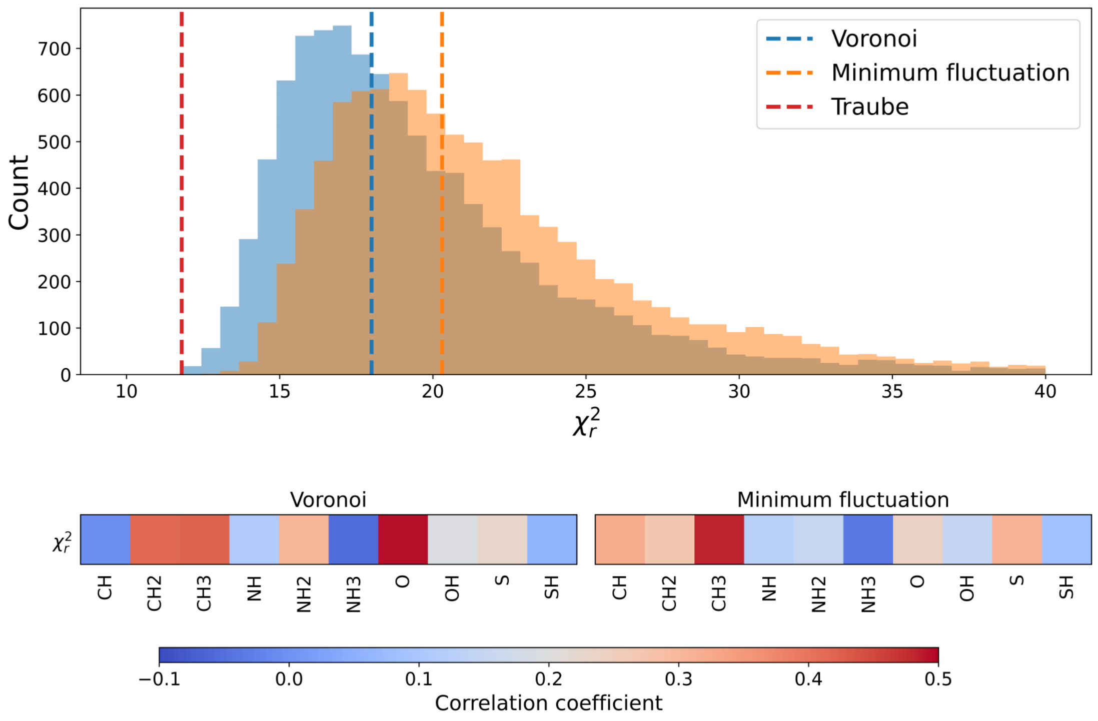

Figure S4
SASDA92: Note how most volumes correlates strongly with the goodness-of-fit χ_r^2 value.

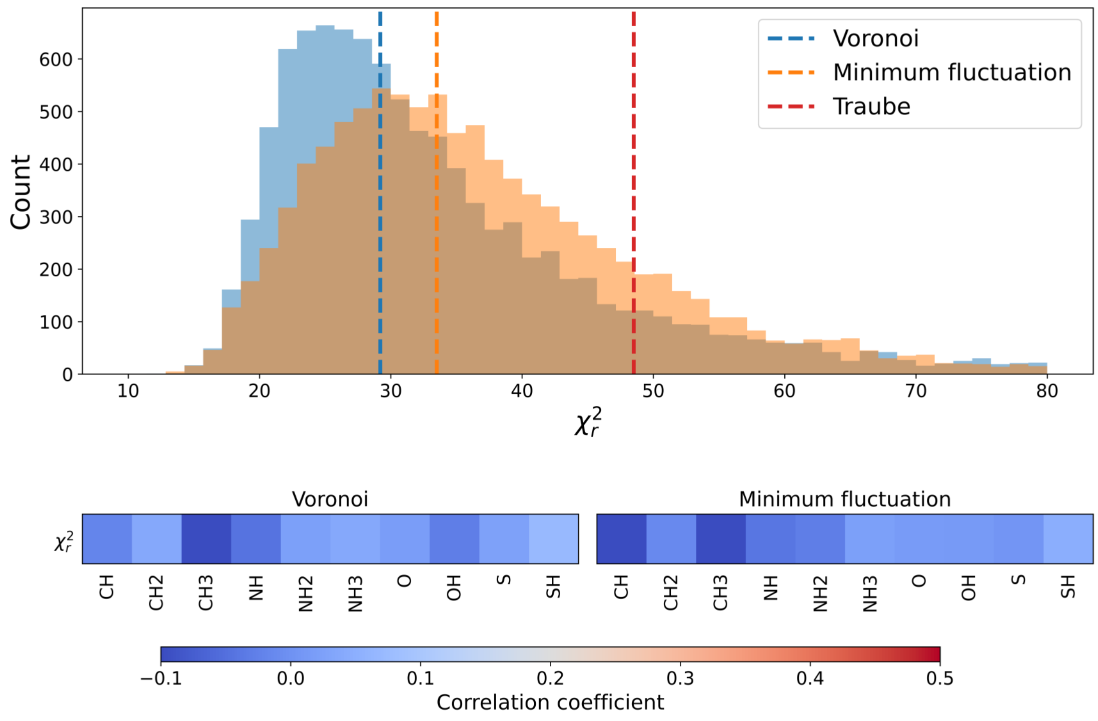

Figure S5
SASDF86: Note the large width of both distributions.

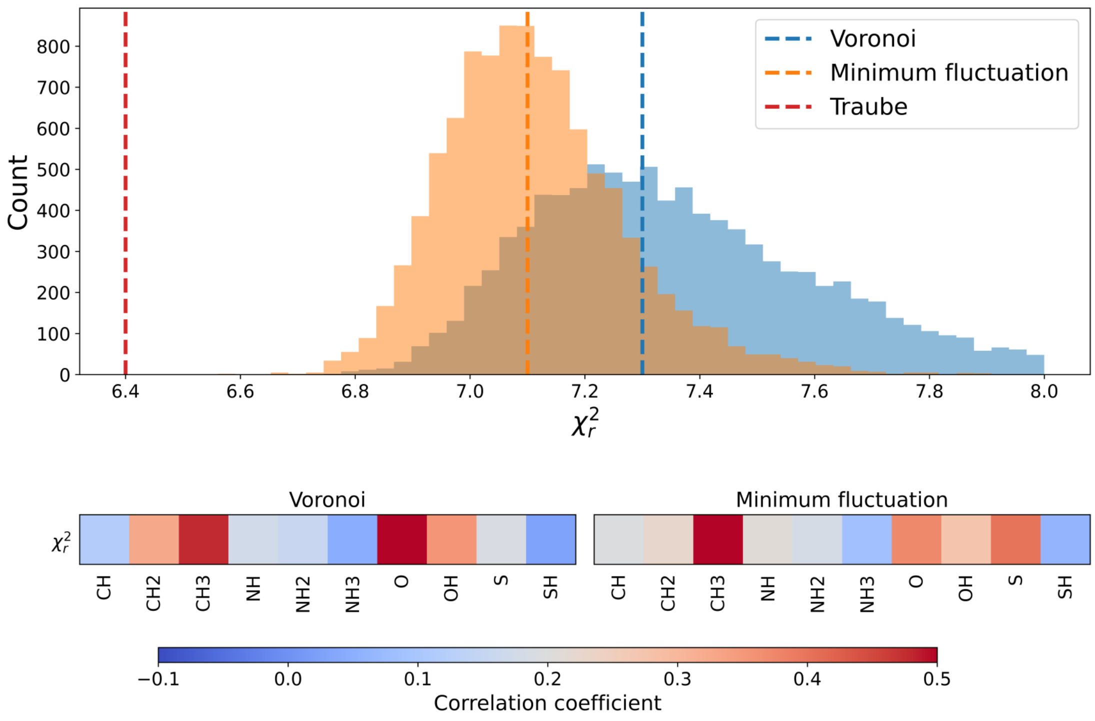

Figure S6
SASDKG4: Note how the minimum fluctuation volumes are better on average in this case. The cause of this shift is unknown. Note also how the Traube volume table is overfitting the data in this case.

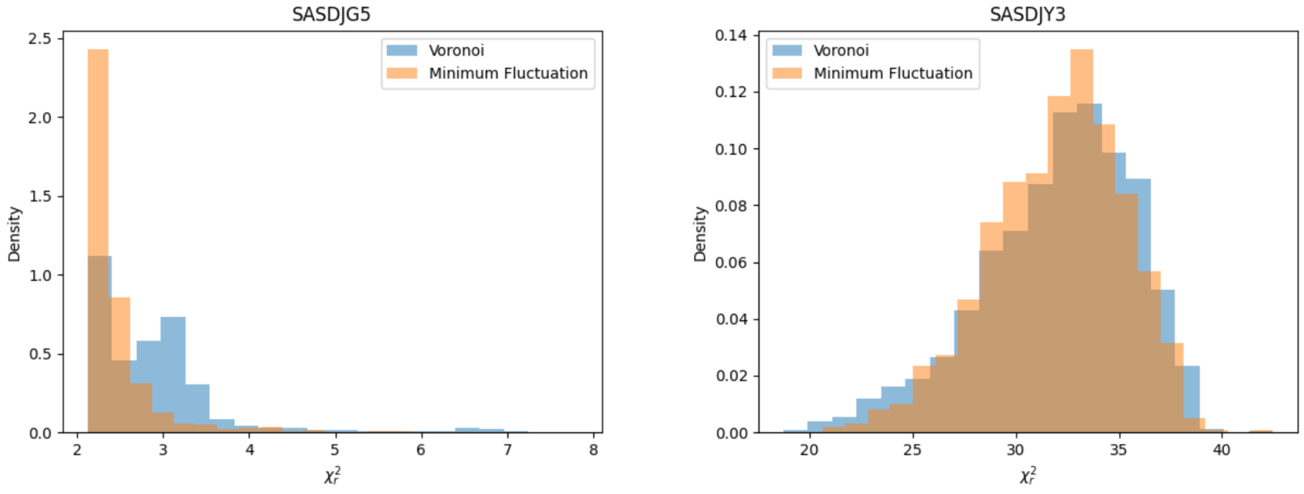

Figure S7
Distributions obtained using `CRYSOL`. Note the similarity to our own results, indicating this is a fundamental issue with the Fraser method itself.

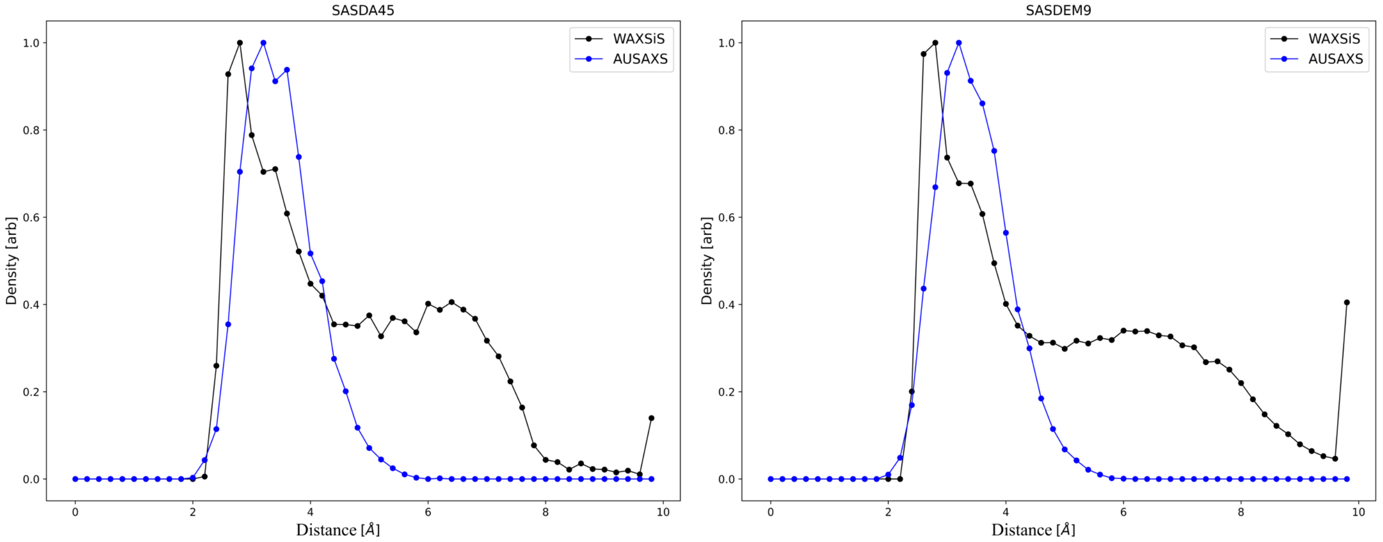

Figure S8
Additional examples comparing the solvent density distribution relative to the protein surface of `WAXSiS` and `AUSAXS`.

1.3. RNA structures

We have also tested our methods on RNA structures, see table S1. We were unable to find high-quality RNA datasets on the SASBDB (Kikhney *et al.*, 2020), so all of the SAXS data used here are heavily oversampled. Curiously, both our grid-based excluded volume method and `CRYSOL` seemingly fails for some of these examples, resulting in the intensity tending to zero in the high-q region. We have excluded `WAXSiS` from these comparisons, as it failed to converge for most of these datasets. This could suggest that perhaps our grid-based method fails because the structures are not physically plausible. It is difficult to analyze the underlying cause without more and higher-quality RNA data to compare against.

Table S1
The full comparison using RNA structures. All structures are available online from the SASBDB repository (Kikhney *et al.*, 2020).

RNA structure	χ^2_{simple}	χ^2_{fraser}	χ^2_{grid}	χ^2_{crysol}	χ^2_{foxs}	χ^2_{pepsi}
SASDAW5	1.04	0.99	0.93	0.88	0.81	0.82
SASDBJ8	4.07	0.68	3.20	0.49	0.46	0.74
SASDBM8	0.75	0.51	0.58	0.71	0.55	0.54
SASDBN8	0.91	0.61	0.81	0.27	0.45	0.45
SASDF29	1.74	1.85	1.46	1.23	1.19	1.44
SASDF39	2.23	0.97	1.07	1.03	1.16	1.09
SASDH44	6.25	6.62	4.62	7.74	3.45	2.93

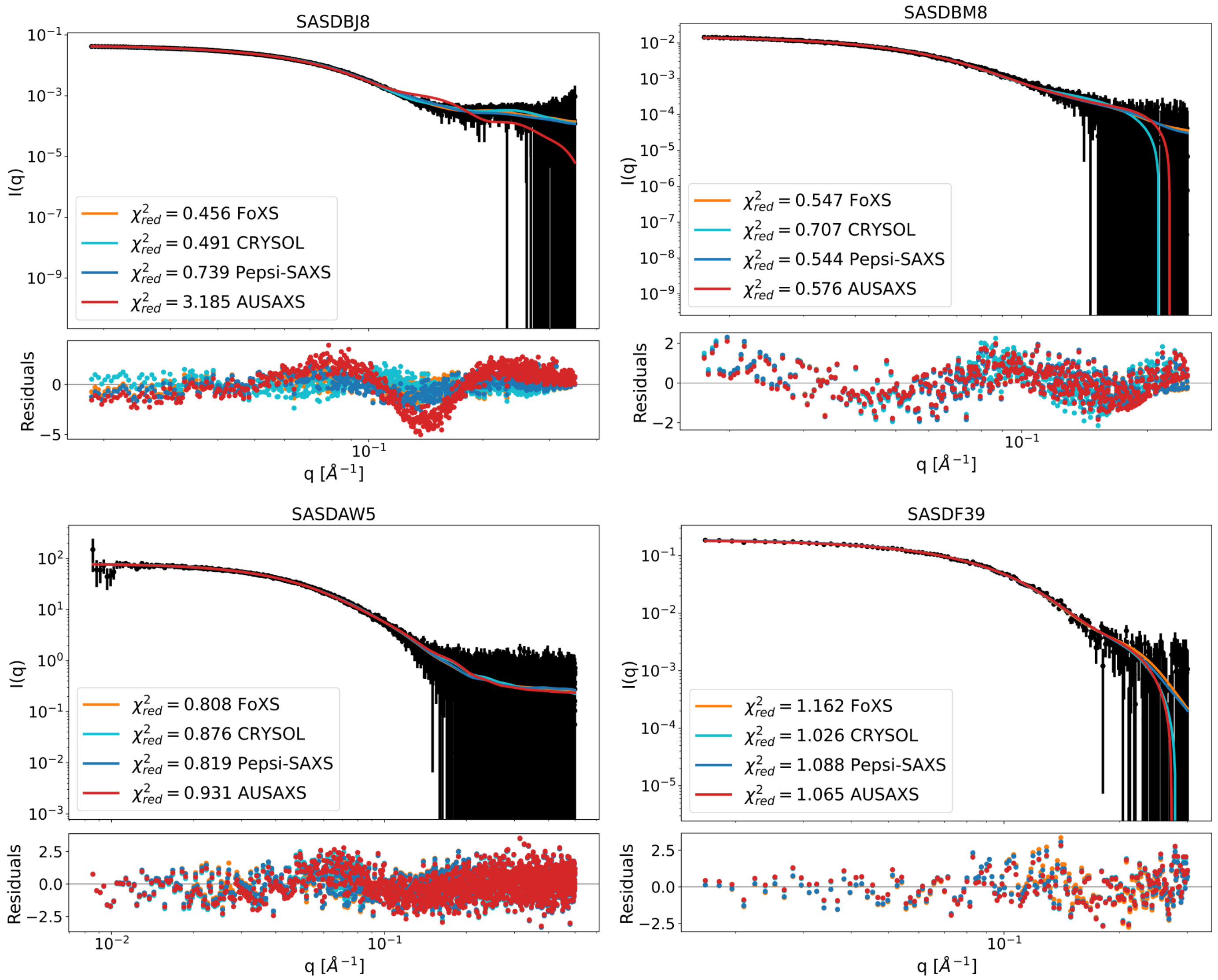

Figure S9

Results for fitting RNA complexes. The grid-based method has been used for the AUSAXS plots. Note how the goodness-of-fit values, χ^2_r, are significantly smaller than expected due to the heavy oversampling at high q.

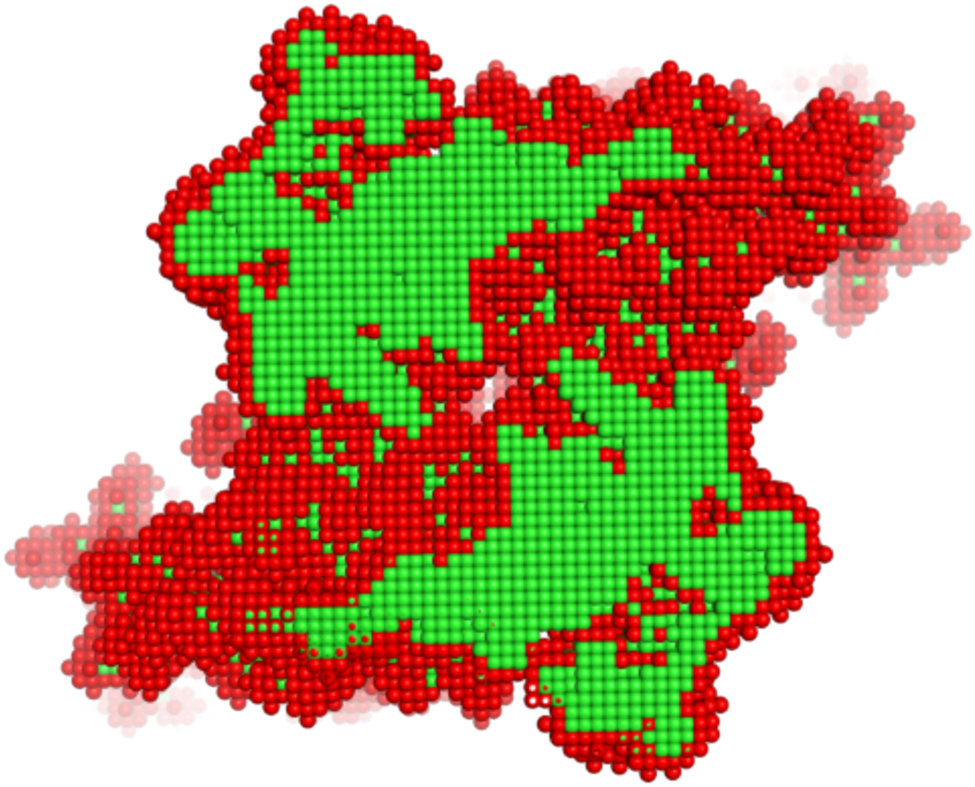

Figure S10
A cross-section of the grid-based excluded volume for the *SASDJG5* structure. First, all grid cells within $\min(2.15, r_{vdw})$ of any protein atom is marked as occupied (green). After this expansion, the surface cells are identified (red), and their form-factors modified to allow for varying their radius by a factor $d \in [0,\ 2]$.

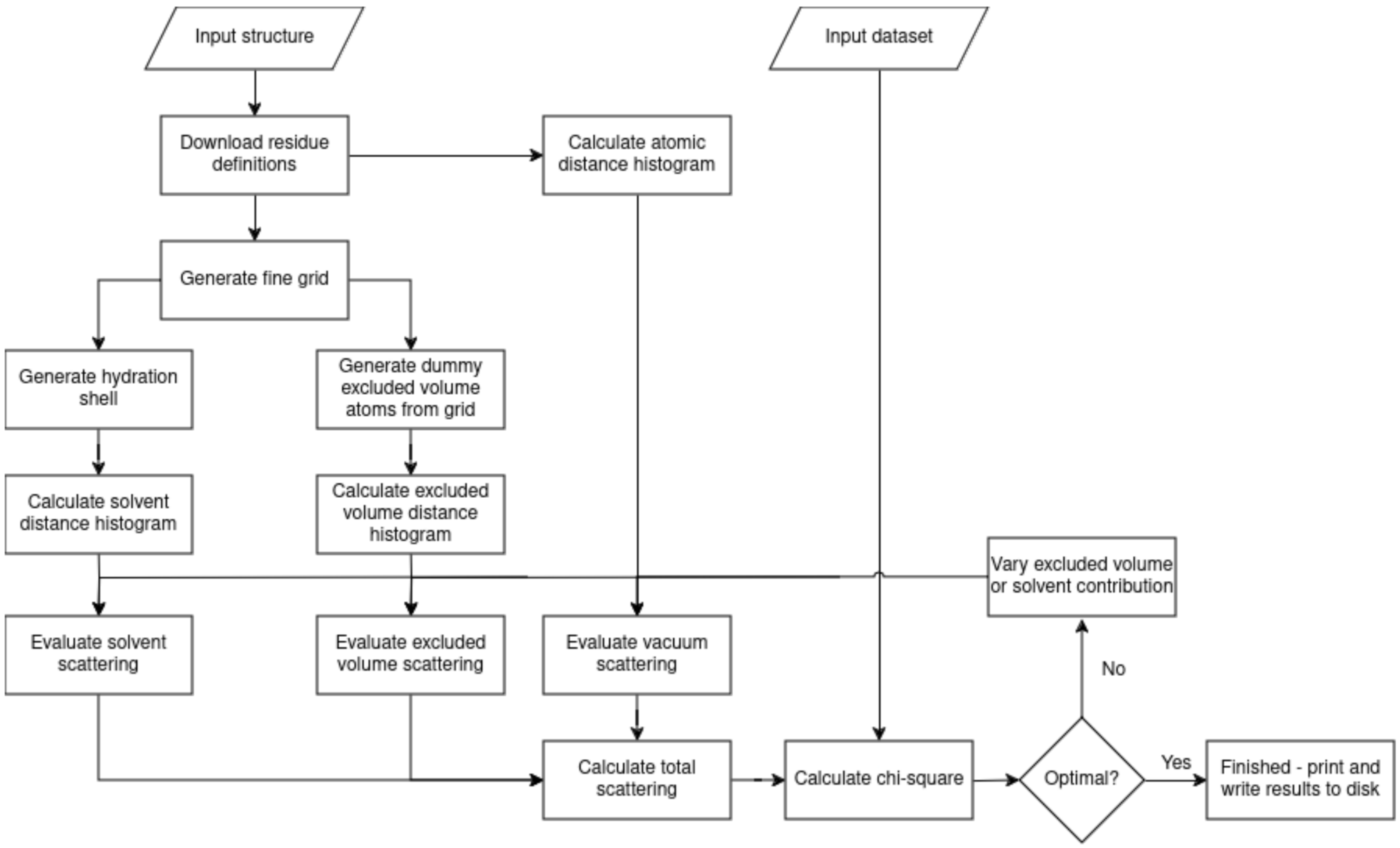

Figure S11
Flowchart illustrating the main points of our method.

Table S2

The test structures, their shape, their mass, and the χ_r^2 values obtained through fitting using the different methods and programs. No correlations could be identified between the shape and accuracy of a given method. All structures are available online from the SASBDB repository (Kikhney *et al.*, 2020).

Structure	Shape	Mass	χ^2_{simple}	χ^2_{fraser}	χ^2_{grid}	χ^2_{crysol}	χ^2_{foxs}	χ^2_{pepsi}	χ^2_{waxsis}
SASDA92	Globular	227	11	28	11	12	15	13	18
SASDAW3	Open elongated structure with hole	76	9.6	14	9.2	13	11	11	10
SASDDD3	Many small domains with holes between	173	6.0	9.3	6.1	7.8	6.5	5.4	6.6
SASDEL9	Cage structure with holes	237	2.5	4.44	1.74	2.17	2.68	1.91	4.04
SASDEM9	Cage structure with holes	438	2.87	1.44	2.63	3.69	3.03	3.5	3.18
SASDF86	Globular. elongated	32	24	6.5	17	16	9.4	5.2	23
SASDJF5	Globular with two protruding parts	22	8.4	6.0	8.0	6.7	7.1	6.1	11
SASDJG5	Globular	68	6.0	6.6	6.6	3.14	2.33	2.79	6.5
SASDJQ4	Ring part with several protruding loops	63	10.0	18	11	9.7	9.8	11	19
SASDJU5	Cage of cylindrical rods	80	2.52	3.52	2.34	5.5	6.0	5.9	8.3
SASDKG4	Two loosely connected globular proteins	75	3.58	3.53	3.54	4.81	4.90	5.3	51
SASDL82	Two connected globular proteins	118	1.74	1.81	1.76	4.71	4.90	5.3	51
SASDMB5	Arc structure with loose loops	65	20	8.2	5.4	4.9	2.47	5.2	17
SASDME4	Elongated structure with some holes	76	1.3	2.46	1.36	3.58	4.54	1.38	6.1
SASDMZ9	Loosely connected domains with flexible linkers	56	15	13	14	11	14	18	26
SASDP39	Globular	58	5.4	7.1	5.9	6.0	11	9.6	67
SASDT75	Rod with loose loops	38	1.81	14	1.56	1.81	1.76	1.91	13
SASDT85	Rod with large loose loops	40	14	20	15	17	13	34	39
SASDT95	Rod with several loops	50	1.8	11	1.99	2.42	1.83	2.30	9.8
SASDA45	Globular. flat closer structure	362	0.71	1.25	0.71	1.85	0.58	0.57	3.63
SASDCQ8	Globular	66	1.05	1.18	1.06	1.09	1.07	1.05	1.14
SASDE35	Globular	46	0.81	0.98	0.79	1.22	0.88	0.83	0.97
SASDE45	Four close-by globular domains	76	1.46	2.52	1.51	1.65	1.19	1.52	2.49
SASDE65	Close-by globular domains	66	1.11	1.62	1.08	1.45	1.22	1.11	1.93
SASDEL8	Elongated structure	126	14	15	13	22	13	13	180
SASDEY4	Globular	14	1.26	1.32	1.32	1.20	1.36	1.08	2.20
SASDFZ3	Prolate. globular	79	0.41	0.61	0.37	0.82	0.69	0.50	3.65
SASDGD2	Globular	33	1.08	1.2	1.07	1.25	1.33	1.22	1.33
SASDHP7	Globular	33	1.4	1.45	1.25	2.02	1.58	1.92	1.46
SASDJG4	Elongated with pending chain	29	1.39	1.56	1.4	2.00	1.42	1.50	5.7
SASDJP5	Elongated cylinder	35	0.99	1.41	0.96	1.85	1.33	1.12	1.57
SASDJQ7	Globular	54	0.88	0.86	0.87	1.14	1.24	1.01	4.91
SASDKH2	Elongated looisely connected	49	1.88	2.02	1.92	2.10	1.95	2.00	1.49
SASDKP2	Globular structure with indent at center	75	1.84	1.99	1.81	2.00	2.26	2.22	3.82
SASDLQ6	Six loosely connected domains	64	0.83	0.83	0.83	1.11	1.09	1.09	0.96
SASDNQ3	Globular. many loosely connected domains	159	31	37	32	110	158	151	574
SASDNV5	Two globular parts. loosely connected	135	223	215	223	253	246	255	369
SASDPB9	Elongated. five loosely connected domains	88	0.59	0.64	0.59	0.78	0.73	069	0.75
SASDPM2	Globular with indents	85	89	95	87	95	96	89	89
SASDQ59	Gloular with indents. loops and a pending end	50	0.92	1.03	0.9	0.90	0.89	0.90	1.11
SASDQN4	Elongated structure	66	1.32	2.34	1.22	1.52	1.15	1.05	1.57
SASDTT4	Globular	12	2.9	2.43	2.19	1.24	2.32	1.71	1.82

```cpp
const double Profile::modulation_function_parameter_ = 0.23;

void Profile::squared_distributions_2_partial_profiles(
const Vector<RadialDistributionFunction>& r_dist) {
    int r_size = r_dist.size();
    init(q_.size(), r_size);

    // precomputed sin(x)/x function
    static internal::SincFunction sf(
    sqrt(r_dist[0].get_max_distance()) * get_max_q(), 0.0001);

    // precompute square roots of distances
    Vector<double> distances(r_dist[0].size(), 0.0);
    for (unsigned int r = 0; r < r_dist[0].size(); r++) {
        if (r_dist[0][r] > 0.0) {
            distances[r] = sqrt(r_dist[0].get_distance_from_index(r));
        }
    }

    bool use_beam_profile = false;
    if (beam_profile_ != nullptr && beam_profile_->size() > 0)
    use_beam_profile = true;

    // iterate over intensity profile
    for (unsigned int k = 0; k < q_.size(); k++) {
        // iterate over radial distribution
        for (unsigned int r = 0; r < r_dist[0].size(); r++) {
            if (r_dist[0][r] > 0.0) {
                double dist = distances[r];
                double x = 0.0;
                if(use_beam_profile) {
                    // iterate over beam profile
                    for (unsigned int t = 0; t < beam_profile_->size(); t++) {
                        // x = 2*I(t)*sinc(sqrt(q^2+t^2)) multiply by 2 because of the
                        // symmetry of the beam
                        double x1 = dist * sqrt((q_[k]*q_[k] + beam_profile_->q_[t]
                        *beam_profile_->q_[t]));
                        x += 2 * beam_profile_->intensity_[t] * sf.sinc(x1);
                    }
                } else {
                    // x = sin(dq)/dq
                    x = dist * q_(k);
                    x = sf.sinc(x);
                }
                // iterate over partial profiles
                for (int i = 0; i < r_size; i++) {
                    // multiply by the value from distribution
                    partial_profiles_[i](k) += r_dist[i][r] * x;
                }
            }
        }
        // this correction is required since we approximate the form factor
        // as f(q) = f(0) * exp(-b*q^2)
        double corr = std::exp(-modulation_function_parameter_ * square(q_(k)));
        for (int i = 0; i < r_size; i++) partial_profiles_[i](k) *= corr;
    }
}
```

Figure S12

Excerpt from the FoXS source code, commit 27926d84dc735a4a978dd495eae3870dfcb37764, showing how form factors are used. Note how only a *single* form factor, corr, is multiplied onto the scattering profile, while it should be the form factor squared. This means that the effective modulation factor is only half of what they describe in their paper. Note that this could potentially still be correct, if the error was also present when the modulation factor was fitted. Code comments are by the FoXS developers.

```cpp
void Profile::sum_partial_profiles(double c1, double c2, bool check_cashed) {
    // precomputed exp function
    static internal::ExpFunction ef(square(get_max_q()) * 0.3, 0.00001);

    if (partial_profiles_.size() == 0) return;

    // check if the profiles are already summed by this c1/c2 combination
    if (check_cashed && fabs(c1_ - c1) <= 0.000001 && fabs(c2_ - c2) <= 0.000001)
    return;

    // implements volume fitting function G(s) as described
    // in crysol paper eq. 13
    double rm = average_radius_;
    // this exponent should match the exponent of g(s) which doesn't have
    // (4pi/3)^3/2 part so it seems that this part is not needed here too.
    // double coefficient =
    // - std::pow((4.0*PI/3.0), 2.0/3.0) * square(rm) * (c1*c1-1.0) /
    // (4*PI);
    double coefficient = -square(rm) * (c1 * c1 - 1.0) / (4 * PI);
    double square_c2 = c2 * c2;
    double cube_c1 = c1 * c1 * c1;

    intensity_ = partial_profiles_[0];
    if (partial_profiles_.size() > 3) {
        intensity_ += square_c2 * partial_profiles_[3];
        intensity_ += c2 * partial_profiles_[4];
    }

    for (unsigned int k = 0; k < size(); k++) {
        double q = get_q(k);
        double x = coefficient * square(q);
        double G_q = cube_c1;
        if (std::abs(x) > 1.0e-8) G_q *= ef.exp(x);
        // double G_q = cube_c1 * std::exp(coefficient*square(q));

        intensity_(k) += square(G_q) * partial_profiles_[1](k);
        intensity_(k) -= G_q * partial_profiles_[2](k);

        if (partial_profiles_.size() > 3) {
            intensity_(k) -= G_q * c2 * partial_profiles_[5](k);
        }
    }
    // cache new c1/c2 values
    c1_ = c1;
    c2_ = c2;
}
```

Figure S13
Excerpt from the FoXS source code, commit 27926d84dc735a4a978dd495eae3870dfcb37764, showing the implementation of the excluded volume fitting. Note how the correct expression from their paper is actually present in a comment, indicating the authors must be aware of the difference. Code comments are by the FoXS developers.

Table S3
The benchmarking data used to generate figure 8 in the main article. All structures marked with a star (*) are available online from the SASBDB repository (Kikhney *et al.*, 2020).

Structure	# atoms	Pepsi-SAXS	FoXS	CRYSOL	Simple	Fraser	Grid
SASDPT4*	1001	20(2)	114(4)	1219(10)	85(4)	1146(7)	2409(122)
SASDPP4*	1004	20(2)	110(4)	1214(13)	81(3)	1136(6)	2382(100)
SASDPS4*	1481	19(2)	159(5)	1253(14)	88(4)	1150(7)	2562(128)
SASDE35*	2917	46(3)	384(10)	1381(9)	116(6)	1195(10)	3153(95)
SASDQ59*	3540	95(4)	552(14)	18207(163)	144(5)	1216(11)	3672(76)
SASDJG5*	4734	70(3)	691(13)	1562(93)	156(6)	1249(6)	4430(66)
SASDME4*	5380	183(10)	975(16)	1620(21)	177(10)	1265(7)	5015(75)
SASDPB9*	6210	437(22)	1713(10)	1664(16)	255(27)	1314(16)	5897(81)
urateox	9436	145(6)	2103(74)	1938(14)	241(8)	1362(13)	10579(155)
SASDPQ4*	9488	228(12)	2158(60)	1956(14)	247(5)	1368(14)	10875(110)
SASDDD3*	10775	82(3)	2671(58)	2052(18)	273(7)	1400(10)	12800(97)
SASDPR4*	12332	402(24)	3279(79)	2169(12)	305(6)	1431(11)	15806(67)
SASDA92*	16068	396(12)	5327(112)	2508(14)	400(6)	1598(8)	24210(75)
SASDEL9*	16640	166(5)	5671(120)	2557(13)	427(8)	1640(11)	27532(123)
A2M_native	43652	2091(40)	34735(495)	4775(27)	1649(8)	4309(44)	174214(757)

References

Kikhney, A. G., Borges, C. R., Molodenskiy, D. S., Jeffries, C. M. & Svergun, D. I. (2020). *Protein Science*, **29**, 66–75. https://onlinelibrary.wiley.com/doi/10.1002/pro.3731

Schaefer, M., Bartels, C., Leclerc, F. & Karplus, M. (2001). *Journal of Computational Chemistry*, **22**, 1857–1879.
